# Genome-wide DNA methylation and transcriptome sequencing analyses of lens tissue in an age-related mouse cataract model

**DOI:** 10.1371/journal.pone.0316766

**Published:** 2025-01-30

**Authors:** Yuzhu Hu, Dongmei Su, Yue Zhang, Yanjiang Fu, Sijia Li, Xiaoya Chen, Xiao Zhang, Shunfei Zheng, Xu Ma, Shanshan Hu

**Affiliations:** 1 Mudanjiang Medical University, Mudanjiang, Heilongjiang, China; 2 Department of Genetics, Health Department, National Research Institute for Family Planning, Beijing, China; 3 Graduate School, Peking Union Medical College, Beijing, China; 4 Daqing Ophthalmology Hospital, Daqing, Heilongjiang, China; 5 Hongqi Hospital of Mudanjiang Medical University, Mudanjiang, Heilongjiang, China; Rutgers: Rutgers The State University of New Jersey, UNITED STATES OF AMERICA

## Abstract

DNA methylation is known to be associated with cataracts. In this study, we used a mouse model and performed DNA methylation and transcriptome sequencing analyses to find epigenetic indicators for age-related cataracts (ARC). Anterior lens capsule membrane tissues from young and aged mice were analyzed by MethylRAD-seq to detect the genome-wide methylation of extracted DNA. The young and aged mice had 76,524 and 15,608 differentially methylated CCGG and CCWGG sites, respectively. The Pearson correlation analysis detected 109 and 33 differentially expressed genes (DEGs) with negative methylation at CCGG and CCWGG sites, respectively, in their promoter regions. Gene Ontology (GO) and Kyoto Encyclopedia of Genes and Genomes (KEGG) functional enrichment analyses showed that DEGs with abnormal methylation at CCGG sites were primarily associated with protein kinase C signaling *(Akap12*, *Capzb*), protein threonine kinase activity (*Dmpk*, *Mapkapk3*), and calcium signaling pathway (*Slc25a4*, *Cacna1f*), whereas DEGs with abnormal methylation at CCWGG sites were associated with ribosomal protein S6 kinase activity (*Rps6ka3*). These genes were validated by pyrosequencing methylation analysis. The results showed that the ARC group (aged mice) had lower *Dmpk* and *Slc25a4* methylation levels and a higher *Rps6ka3* methylation than the control group (young mice), which is consistent with the results of the joint analysis of differentially methylated and differentially expressed genes. In conclusion, we confirmed the genome-wide DNA methylation pattern and gene expression profile of ARC based on the mouse cataract model with aged mice. The identified methylation molecular markers have great potential for application in the future diagnosis and treatment of ARC.

## Introduction

A cataract is partial or complete opacity of the lens of the eye. Cataracts are an acquired conditions among adults, and the most prevalent type is age-related cataract (ARC) [1]. The lens is formed from ectodermal tissue and contains lens epithelial cells (LECs) that produce lens fibers throughout life, and becomes more compact and thicker with age [2]. Because LECs retain intact nuclei and organelles, they help to maintain lens metabolic homeostasis [3]. LECs produce cysteine and glutathione to maintain the lens environment in the presence of harmful external stimuli [4]. Cataract surgery has improved, and most patients recover vision quickly. Despite this, cataracts remain a serious public health issue and will become more important as the global population and life expectancy increase [5].

Epigenetics provides a new explanation for ARC [6]. Epigenetics pertains to heritable alterations in genomic structure that occur without any changes in the DNA sequence. Investigations of epigenetic mechanisms encompass DNA methylation, histone modifications, and microRNAs. DNA methylation is a major mechanism for epigenetic regulation of genes [7]. Changes in the expression of DNA repair genes have been linked to the pathogenesis of ARC [8]. Human lens development requires the expression of multiple important genes, and DNA methylation regulates the expression of many development-related genes. Environmental exposures also alter DNA methylation patterns. Therefore, DNA methylation modifications may be associated with the pathogenesis of cataracts that are influenced by age and environmental factors, as well as lens development [7]. Abnormal methylation is an important characteristic of epigenetics and a crucial factor that can lead to cataracts and other ocular disorders [6]. Therefore, a comprehensive exploration of the particular mechanism of DNA methylation in the progression of ARC is of great significance.

**Fig 1:**
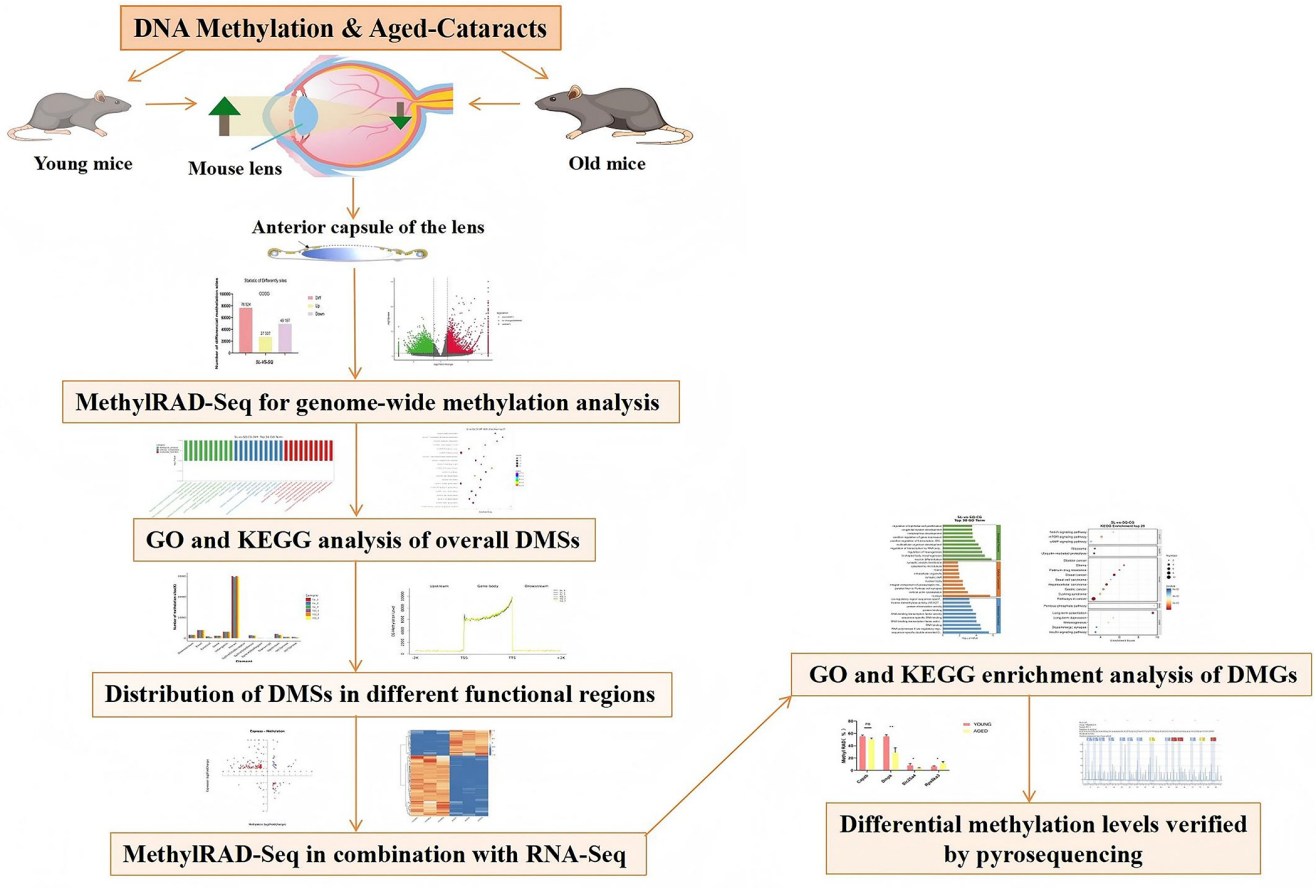
Experimental procedure and design analysis. DNA samples were collected from the anterior lens capsule membrane tissues of three young mice (8 months old) and three aged mice (20 months old) for genome-wide methylation analysis. Subsequently, GO and KEGG analyses were conducted on the DMGs at CCGG and CCWGG sites. Next, the distribution of DNA methylation sites in different functional regions was explored. Then, a joint analysis of methylation and previous transcriptome data was carried out. After that, GO and KEGG enrichment analyses were performed on the DEGs with differential methylation at CCGG and CCWGG sites in the promoter region. Finally, pyrosequencing was used to validate the candidate genes with five samples from the young group (normal group,8 months old) and five samples from the natural aging group (ARC group,20 months old), thereby confirming the accuracy of the methylation sequencing data.

In this study, the experimental procedure and design analysis is shown in **Fig 1.** Specifically, we first identified methylation across the entire genome in the anterior lens capsule membranes of young and aged mice by MethylRAD-seq. Then, we used the previously published transcriptome sequencing data for the same disorder in the same model [9] to identify differentially expressed genes (DEGs) with negative methylation in their promoter regions. Next, we performed Gene Ontology (GO) and Kyoto Encyclopedia of Genes and Genomes (KEGG) functional enrichment analyses on the DEGs with negative methylation. We verified gene methylation levels by pyrophosphate sequencing to identify novel therapeutic targets using bioinformatics tools.

## Materials and methods

### 1. Mice and isolation of the lens anterior capsule membrane

We raised 40 wild-type young mice (8 months old) at 22 ± 2°C, 55 ± 5% humidity, and 12-h/12-h light/dark cycle. The lenses of the young mice were observed and found to be transparent. Ten of the young mice were chosen randomly as the normal control group. To simulate the occurrence of cataracts in the natural aging state of individuals, we raised 40 mice for 20 months under the same conditions. The lenses of the aged mice were observed. We found that the cataracts gradually became turbid with increasing age using a slit lamp. We selected 10 mice with cataracts that had Grade 3 turbidity as the ARC group [9].

The experimental mice were anesthetized with 2.0% isoflurane inhalation and samples were taken. Then, the mice were euthanized by CO_2_ asphyxiation. We collected DNA samples from the anterior lens capsule membrane tissues (only contain LECs) of three young mice (8 months old) and three aged mice (20 months old) for genome-wide methylation analysis as described previously [10–13]. For young mice, the number of anterior lens capsule samples is three, that is, n = 3. They are in the healthy group and are named SQ-1, SQ-2, and SQ-3 respectively. For naturally aging mice, the number of anterior lens capsule samples is also three. n = 3 is the disease group, that is, suffering from cataracts. They are named SL-1, SL-2, and SL-3 respectively.All the animal experiments were approved by the Animal Protection and Use Committee of Mudanjiang Medical University (No. 20220228–41) and were in line with the ARVO Statement for Use of Animals in Ophthalmic and Vision Research. The experimental procedure also complied with the National Institutes of Health Guidelines for the Care and Use of Laboratory Animals.

### 2. DNA extraction and MethylRAD experimental process

Genomic DNA from the anterior lens capsule membrane tissue was extracted using the TIANamp Micro DNA Kit according to the manufacturer’s instructions. Libraries were constructed following the guidelines established by Wang et al. [14]. Four units of the methylation-modifying endonuclease FspEI (New England BioLabs, cat. no. R0662L) were used to break down 200 ng of DNA at 37°C for 4 h. Then, the DNA was joined to an aptamer. To initiate the ligation procedure, 5 μl of fragmented DNA was added to 10 μl of ligation master mixture (800 units of T4 DNA ligase (New England Biolabs), 1 mM of adenosine triphosphate (ATP), and 0.2 μM of each of the two aptamers). Subsequently, the amplified PCR product was purified by 8% polyacrylamide gel electrophoresis. The approximately 100-base pair band was extracted, and the DNA was spread at 4°C for 12 h, then rinsed off the gel using nucleic acid-free water. The products were amplified again by PRC. The PCR products were purified using a QIAquick PCR Purification Kit (Qiagen cat. No.28104.), eluted with 15 μl of purified water, and measured by Qubit (Qubit 2.0 fluorometer (Invitrogen)). to quantify the product. The purified PCR product were sequenced on an Illumina Nova PE150 platform (Shanghai OE Biotechl Co.,Ltd.,Shanghai,China). FspEI has different preferences for sequences on each side of methylation-modified cytosines, and therefore the types of sites analyzed by FspEI are different. For a ^m^CG site, FspEI analyzes only the CCGG site. For a ^m^CHG site, where H is either A, C, or T, when H is base C, the data processing recognizes the site as CCGG. Therefore, FspEI only provides results for CCAGG and CCTGG for the ^m^CHG site, denoted by CWG, where W is either A or T.

### 3. Alignment and quality control

First, adapter trimming and quality filtering were conducted on the raw reads. A custom Perl script was used to remove reads with >8% ambiguous bases (N), low-quality reads (15% of the bases had a Phred quality score <30), or reads that lacked enzyme restriction sites. Subsequently, Bowtie 2 (v.2.3.4.1) [15] was used to align the reads that contain enzyme restriction sites (enzyme reads) to the mouse genome (GRCm39) (https://ftp.ncbi.nlm.nih.gov/genomes/all/GCF/000/001/635/GCF_000001635.27_GRCm39/GCF_000001635.27_GRCm39_genomic.fna.gz) [16] using the default settings.The default settings are as follows:the parameter is set as:—no-unal, that is, records that do not meet the alignment requirements are not output. Since the reference sequence has repeat regions, reads in the repeat regions can be aligned to multiple positions, making it difficult to determine the actual position of the methylation site. After random alignment, reads with multiple alignments are deleted. Obtain Clean Reads that are aligned to a unique position on the reference sequence. Perform electronic digestion on the genome and count the number of various enzyme digestion sites on each chromosome (or scaffold). Next,The distributions of cytosine methylation sites across the mouse chromosomes and other functional genomic regions were analyzed. The normalized read depth metric RPM (reads per million) was used to obtain the relative expression level of each methylation site (CpG).The sequencing depth of methylation tags can reflect the methylation level of sites. Since the FspEI enzyme can only recognize methylated sites and cut them, theoretically, the higher the methylation level of this site, the more tags are cut off. Therefore, the higher the tag depth, the higher the methylation level. However, the depth needs to be normalized by the amount of sequencing data of the sample. Therefore, there is the RPM method, that is RPM = [(read coverage at each site/number of high-quality reads in the library) × 1,000,000]. The R package DESeq [17] was used to assess changes in the methylation level in accordance with the sequencing depth of each site within the relative quantitative outcomes of methylation.The sequencing data were used to determine the fold change (log2FC >1), and *P*-value <0.05 among diverse sites was considered to be statistically significant. (GO) and KEGG functional enrichment analyses were carried out on the differentially methylated genes (DMGs).

### 4. GO and KEGG functional enrichment analyses

The functional enrichment analyses were performed using Python scripts to identify methylation-related GO(http://geneontology.org/) terms and KEGG (http://www.genome.jp/kegg/) pathways associated with the DMGs. The hypergeometric distribution was used to determine the significance of the annotations of the DMGs to each GO term and each KEGG pathway, against the complete list of protein coding genes in the mouse genome as the background.

### 5. DNA methylation combined with transcriptome sequencing

To refine the methodology, transcriptome sequencing was performed using a NEBNext® Ultra™ II Directional RNA Library Prep Kit for low-input samples. Library quality was assessed with a 2100 Bioanalyzer, and sequencing was performed on an Illumina HiSeq X Ten platform to obtain paired-end reads. HISAT2 was used to align the reads to the reference mouse genome, and FPKM values were used to normalize gene expression. The R package DESeq identified DEGs with *P* <0.05(the *P* value here is corrected by FDR) and fold change >2. To explore the relationship between gene expression and DNA methylation, we performed a Pearson correlation analysis between DEGs with abnormal methylation at CCGG and CCWGG sites in their promoters and their mRNAs. Subsequently, using a *P*-value threshold of <0.05(the *P* value here is corrected by FDR) and a correlation coefficient of < -0.8, we integrated the analyses of DMGs and DEGs within promoter regions.

### 6. Pyrosequencing verification

DNA was extracted using a TIANamp Micro DNA Kit. Methylation was carried out using a Qiagen EpiTect Bisulfite Kit (Qiagen59104), ensuring a comprehensive and standardized approach. Primer design was performed using PyroMark Assay Design 2.0. After PCR amplification, pyrosequencing was conducted on an advanced PyroMark Q48 Autoprep platform (Qiagen). The methylation profiles of individual sites were systematically analyzed using the proprietary PyroMethylation Analyzer software, thereby facilitating an automated and efficient assessment. The primer sequences used in this study are listed in **Table 1**.

**Table 1:** Primer sequences and product lengths in each assay.

Gene	Primer Sequence (Forward, Reverse, Sequencing)	Length(bp)
Capzb-F	AGGGAGGGTTTTAGAAAGTTATT	200bp
Capzb-R	TTACCCACAATACACTTCCCTAAA	
Capzb-S	TTGTATGGGAATAGATATAAAA	
Dmpk-F	GAGAATAAGAGGAGAAAGGTGGATTAG	91bp
Dmpk-R	AACAACAACTCCAAAACCTTTAAATA	
Dmpk-S	AGTATTTGGGTTTTTGTTAG	
Rps6ka3-F	GAAGGGGTAGGAGGTGGAAA	163bp
Rps6ka3-R	CCCCACTTCCCCTCACAACATAAC	
Rps6ka3-S	GTAGGAGGTGGAAAAG	
Slc25a4-F	GTTGGGTTGGTTTTTGGATAGT	96bp

### 7. Statistical analysis

Data are presented as the mean ± standard deviation for at least three experiments. GraphPad Prism 8 software was used to process the data. To evaluate the differences between the control and ARC groups we used a t-test. For multiple comparisons, one-way analysis of variance (ANOVA) was used to detect significant differences. Differences were considered statistically meaningful for *P*-values <0.05.And the *P*-values are all *P*-values after FDR correction.

## Results

### 1. MethylRAD-seq quality control

**Fig 2:**
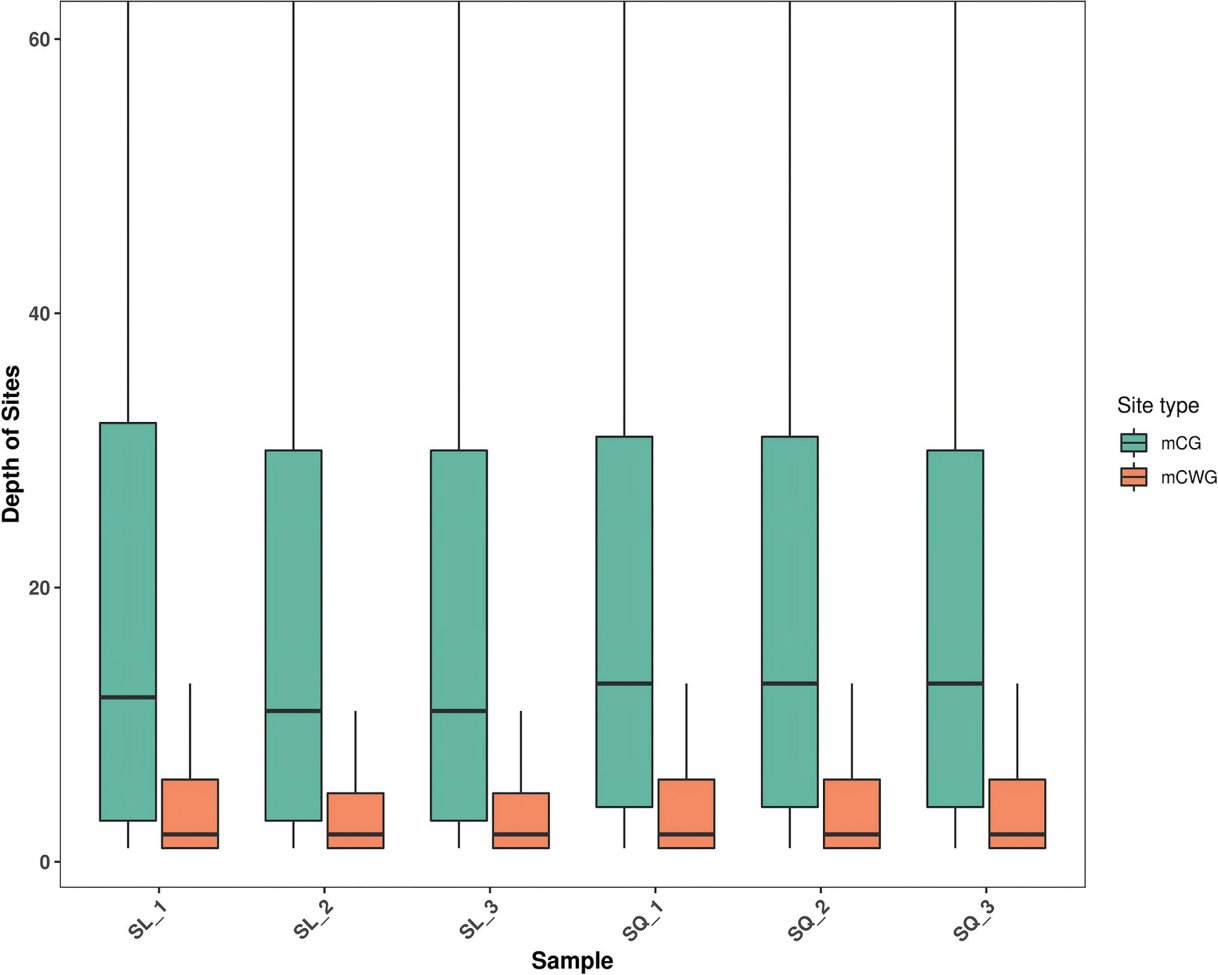
Depth of coverage statistics for methylation sites. Green color indicates a box plot representing the sequencing depth of CCGG methylation sites in each sample. Orange color stands for the boxplot of the sequencing depth of CCWGG methylation sites in every sample.

We performed methylation sequencing by MethylRAD-seq, which specifically recognizes alterations in cytosine C5 methylation on DNA using the FspEI enzyme. MethylRAD-seq systematically performs digestion at predetermined intervals, and selectively gathers DNA fragments of 13–17 base pairs that are methylated across the entire genome. This process facilitates both qualitative and relative quantitative assessments of methylation sites. After re-filtering the raw data, paired-end sequencing reads are mapped following the criteria outlined in the Materials and Methods section, to obtain diverse read types, including enzymatic and clean reads for each individual sample as shown in **Table 2**. Each sample yielded an average of approximately 78.02 million reads. The FspEI treatment resulted in an average of 32.25 million enzymatic reads and 27.94 million cleaned reads (**Table 2**). The depth of sequencing coverage for MethylRAD-seq sites, specifically CCGG and CCWGG motifs, for each sample is visualized by the box plots in **Fig 2**. The samples contained an average of 830,000 CCGG and 380,000 CCWGG motifs. The mean coverage depth was 21 for CCGG and 7.97 for CCWGG (**Table 3**). The enzymatic reads for each sample were aligned against the established reference sequences. To ensure the reliability of the methylation status, only sites with a sequencing depth of three-fold or more were considered validly methylated [18,19]. The sequencing reads that satisfied these prerequisites were used for subsequent analyses.

**Table 2:** Statistical table on changes in data volume.

Sample	Raw Reads	Norm Reads	Adapter Reads	Enzyme Reads	Range Reads	Clean Reads	Percent
SL_1	83033801	61128449	60534408	34656532	31503675	30395078	49.72%
SL_2	69481889	60936752	60308475	33432782	30130714	28994912	47.58%
SL_3	96242834	61229760	60620405	32923888	29528455	28481767	46.52%
SQ_1	65563461	61567057	60584303	31185676	28008463	26894267	43.68%
SQ_2	73526131	60450114	59438500	30808513	27676897	26595900	44.00%
SQ_3	80308732	60602711	59546888	30539711	27343383	26320986	43.43%

**Table 3:** Statistics on the depth of coverage for methylation sites.

Sample	CG_Site_Num	CG_Site_Depth	CWG_Site_Num	CWG_Site_Depth
SL_1	851133	22.5	350184	8.71
SL_2	843258	21.36	371804	7.98
SL_3	836521	21.07	385816	7.76
SQ_1	837385	21.05	387132	7.91
SQ_2	832443	20.88	391180	7.81
SQ_3	826643	20.71	404810	7.67

### 2. Genome-wide methylation analysis by MethylRAD-seq

**Fig 3:**
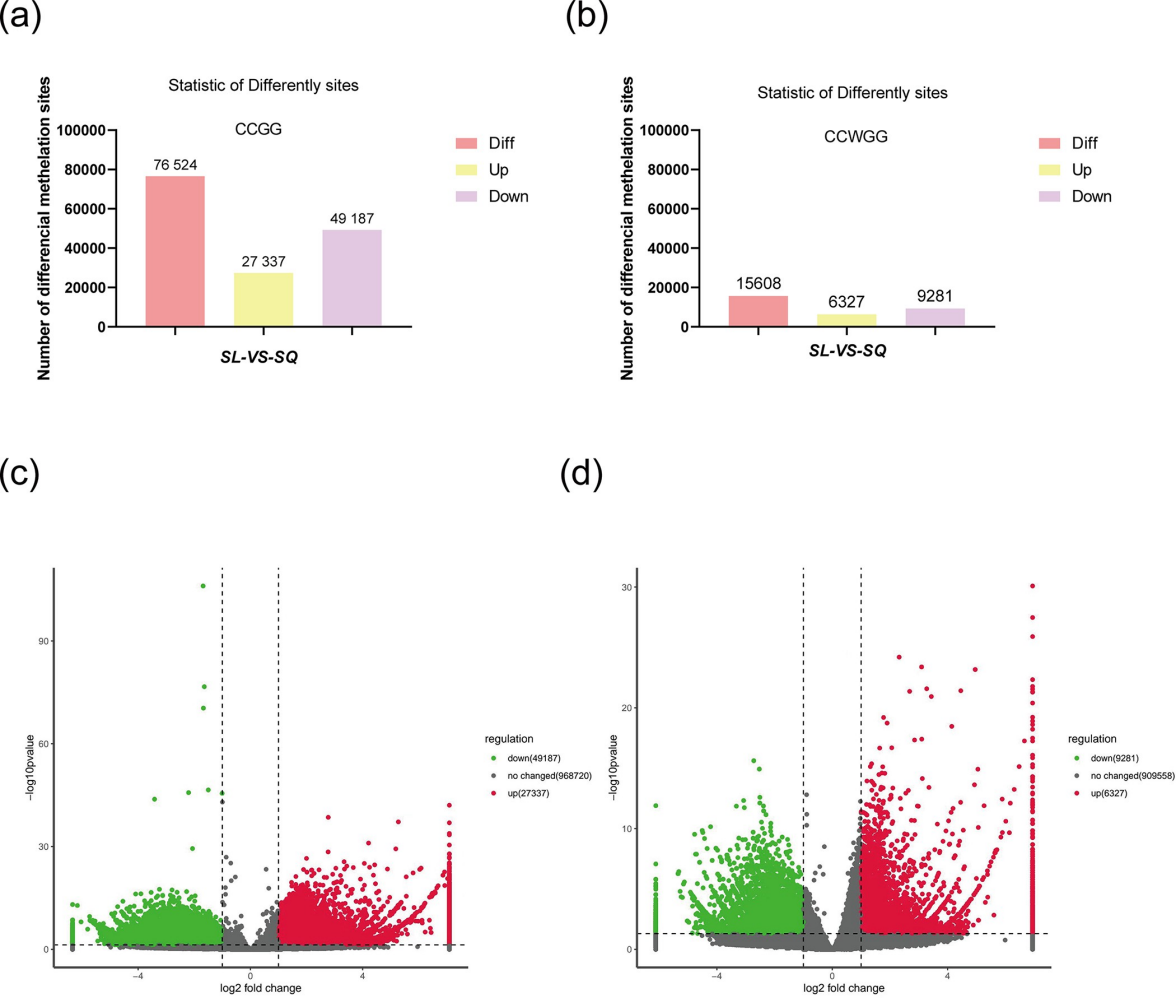
Genome-wide methylation analysis of the anterior lens capsule membranes of young and old mice by methyl-rad-seq. **(**a) Histogram of DMSs at CCGG sites. Red: Total number of DMSs; yellow: Hypermethylated; purple: Hypomethylated. (b) Histogram of DMSs at CCWGG sites. Red: Total number of DMSs; yellow: Hypermethylated; purple: Hypomethylated.(c) Volcano plot of DMSs at CCGG sites. Gray: Non-DMS; red: Hypermethylated; green: Hypomethylated. (d) Volcano plot of DMSs at CCWGG sites. Gray: Non-DMS; red: Hypermethylated; green: Hypomethylated.All DMSs at CCGG and CCWGG sites can be found in **S1** and **S2** Tables.

To identify differentially methylated sites(DMSs), depth information (base mean estimated expression) was normalized using DESeq software, differential multiplicity was determined, and the number of reads was checked for significance using the negative binomial distribution test. Credible methylation sites with *P* <0.05 and multiplicity of difference >2 are shown in **Fig 3**. The tissue samples from young and aged mice had 76,524 and 15,608 DMSs at CCGG and CCWGG sites (**S1 and S2 Tables**), respectively; 27,337 were hypermethylated and 49,187 were hypomethylated at the CCGG sites (**Fig 3A and 3C**), and 6,327 were hypermethylated and 9,281 were hypomethylated at the CCWGG sites (**Fig 3B and 3D**).

### 3. GO and KEGG analyses of the differentially methylated genes at CCGG and CCWGG sites

**Fig 4:**
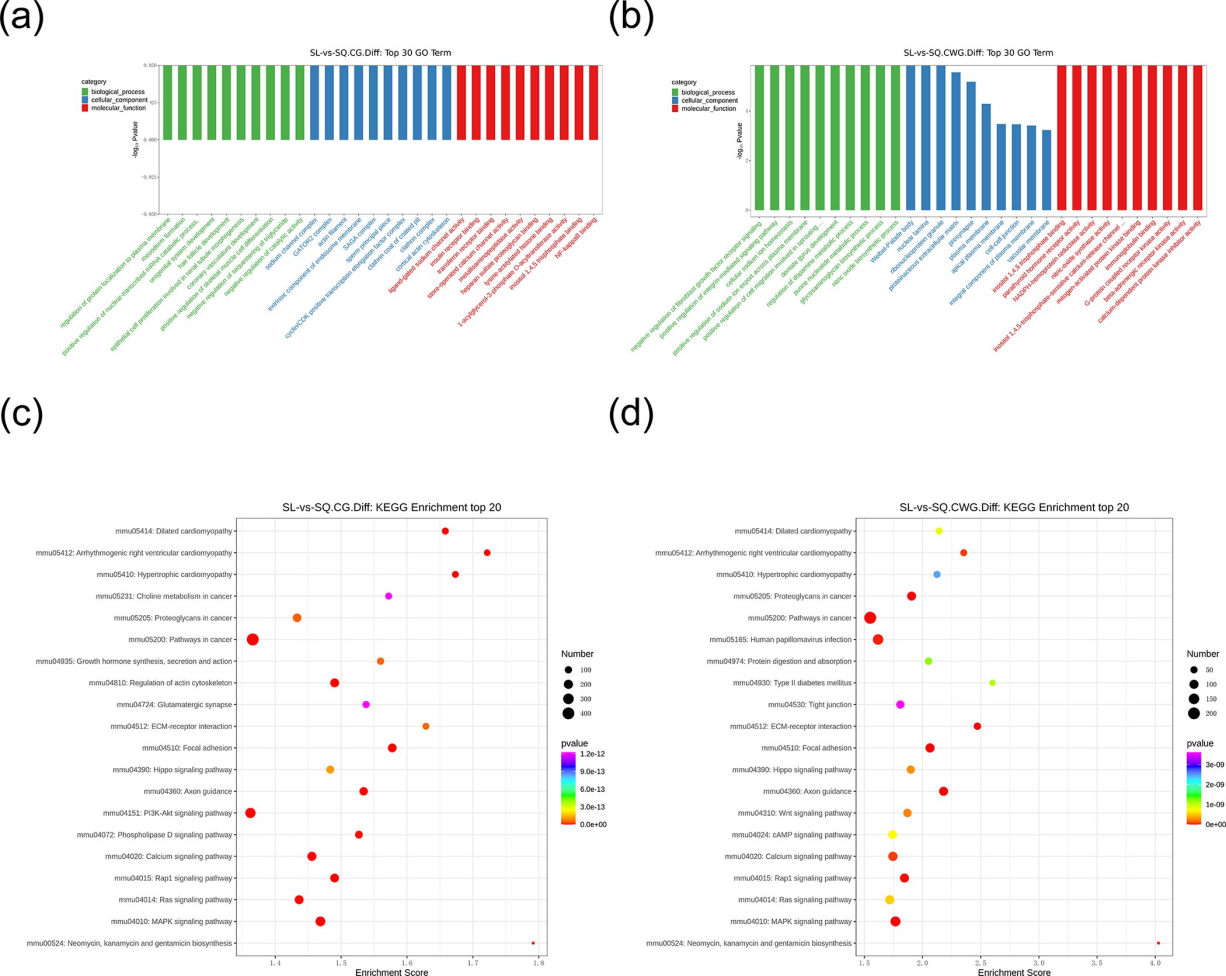
GO and KEGG analyses of DMGs with differentially methylated CCGG and CCWGG sites. (a) Histogram of GO functional classification of (DMGs where differentially methylated CCGG sites are located. In the figure, the horizontal axis represents the GO entry name, which is actually the description of the gene function; the vertical axis is the -log10P value, and the larger the value, the higher the significance of the enrichment of this functional category in the gene set. The GO enrichment analysis was used to screen out the top 30 GO terms. Among these three functional classifications, the GO terms with the number of genes related to the corresponding differentially methylated sites exceeding 2 were selected. Then, they were sorted in descending order according to the -log10 *P* value corresponding to each term. Finally, 10 terms were taken from each classification.(b) Histogram of GO functional classification of DMGs where differentially methylated CCWGG sites are located.The results of GO analysis show the DMGs related to molecular functions (labeled in red), cellular components (labeled in blue), and biological processes (labeled in green).(c) Bubble chart of KEGG enrichment analysis of DMGs where differentially methylated CCGG sites are located.The bubble chart of the top 20 KEGG enrichment analysis (screening the pathway entries with the number of corresponding differentially expressed genes greater than 2 and sorting them from large to small according to the -log10 *P* value corresponding to each entry). In the figure, the X-axis Enrichment Score is the enrichment score, the larger the bubble, the more differential protein-coding genes the entry contains, the bubble color changes from purple-blue-green-red, and the smaller the enrichment pvalue value, the greater the significance. The Y-axis is the descriptive information of the enriched pathways.(d) Bubble chart of KEGG enrichment analysis of DMGs where differentially methylated CCWGG sites are located.

We carried out a comprehensive GO functional enrichment analysis for the three main categories of GO, namely biological process, cellular component, and molecular function.For the entries that are significantly enriched (*P* < 0.05) in each category, they are all ranked according to their *P*-values. It should be noted that the larger the -log10*P* value, the higher the significance of the enrichment of this functional category in the gene set. During the GO enrichment analysis process, we screened out the top 30 GO entries. The specific operation is to first select the GO entries with the number of genes related to the differentially methylated sites greater than 2 in these three functional classifications, and then sort them from large to small according to the -log10 *P* value corresponding to each entry, and finally select 10 entries from each classification. DMGs at CCGG sites were annotated with 106,297 GO terms; 60,246 under biological process, 36,911 under cellular component, and 9,140 under molecular function. DMGs at CCWGG sites were annotated with 46,895 GO terms; 27,346 under biological process, 36,911 under cellular component, and 9,140 under molecular function. The terms assigned to the top 10 DMGs in each category were selected for further analysis. For the CCGG-based DMGs, the biological process terms were primarily linked to regulation of protein localization to the plasma membrane (e.g., *Vamp8*, *Cdkl5*, *Pik3r2*), the cellular component terms were mainly associated with actin filament (e.g., *Tek*, *Capzb*, *Rac2*), and the molecular function terms was mainly associated with store-operated calcium channel activity (e.g., *Trpc7*, *Trpc1*, *Trpm8*) as shown in **Fig 4A**. For the CCWGG-based DMGs, the biological process terms were mainly associated with cellular sodium ion homeostasis (*Atp1b3*, *Atp1b1*, *Slc8a3*), the cellular component terms was mainly associated with the Weibel-Palade body (*Col6a2*, *Vwf*, *Bmper*), and the molecular function terms were mainly related to inositol 1,4,5 trisphosphate binding (*Plekha7*, *Itpr3*, *Itpr2*, *Itpr1*) as shown in **Fig 4B**.

To predict the pathways in which DMGs may participate, we performed KEGG enrichment analysis (**Fig 4C and 4D**). Top 20 of KEGG Enrichment Analysis: Screen the pathway entries with the number of corresponding differentially methylated sites genes greater than 2 and sort them from large to small according to the -log10P value corresponding to each entry. In the figure, the Enrichment Score on the X-axis is the enrichment score. The larger the bubble, the more differential protein-coding genes the entry contains. The bubble color changes from purple to blue, green, and red. The smaller the enrichment *P* value, the greater the significance. The Y-axis is the descriptive information of the enriched pathways. For the CCGG-based DMGs, the enriched pathways were primarily biochemical signaling pathways, notably the mitogen-activated protein kinase (MAPK) signaling cascade (e.g., *Mapk1*, *Mapk3*, *Tek*, *Rac2*), the Ras-related protein 1(Rap1) signaling pathway (e.g., *Actb*, *Pik3r2*, *Tek*, *Actg1*), the calcium signaling pathway (e.g., *Adcy1*, *Adora2a*, *Fgf9*, *Tek*, *Slc25a4*), the PI3K-Akt signaling pathway (e.g., *Akt1*, *Tek*, *Akt2*), the Ras signaling pathway (e.g., *Map2k1*, *Tek*, *Pak1*), and the Hippo signaling pathway (e.g., *Gsk3b*, *YAP1*) as shown in **Fig 4C**. For the CCWGG-based DMGs, the enriched pathways were predominantly biochemical signaling pathways, specifically the MAPK signaling pathway (e.g., *Mapk1*, *Mapk3*), the Rap1 signaling pathway (e.g., *Mapk1*, *Mapk3*, *Tiam1*, *Rap1a*), the calcium signaling pathway (e.g., *Cacna1f*, *Itpr1*, *Itpr2*, *Itpr3*, *Slc8a3*), the Wnt signaling pathway (e.g., *Ror1*, *Wnt7b*), the Hippo signaling pathway (e.g., *Yap1*, *Wnt7b*), the Ras signaling pathway (e.g., *Mapk1*, *Mapk3*), the cAMP signaling pathway (e.g., *Map2k1*, *Atp1b3*, *Atp1b1*, *Edn2*), and Type II diabetes mellitus (e.g., *Hk1*, *Mafa*) as shown in **Fig 4D**. Next, we conducted a combined analysis of individual DMGs that were chosen based on the GO and KEGG analyses. The selected consensus CCGG-based DMGs included *Rac2*, *Tek*, and *Pik3r2*, and the selected consensus CCWGG-based DMGs included *Atp1b3*, *Atp1b1*, *Itpr1*, *Itpr2*, and *Itpr3*.

### 4. Distribution of DNA methylation sites in diverse functional regions

**Fig 5:**
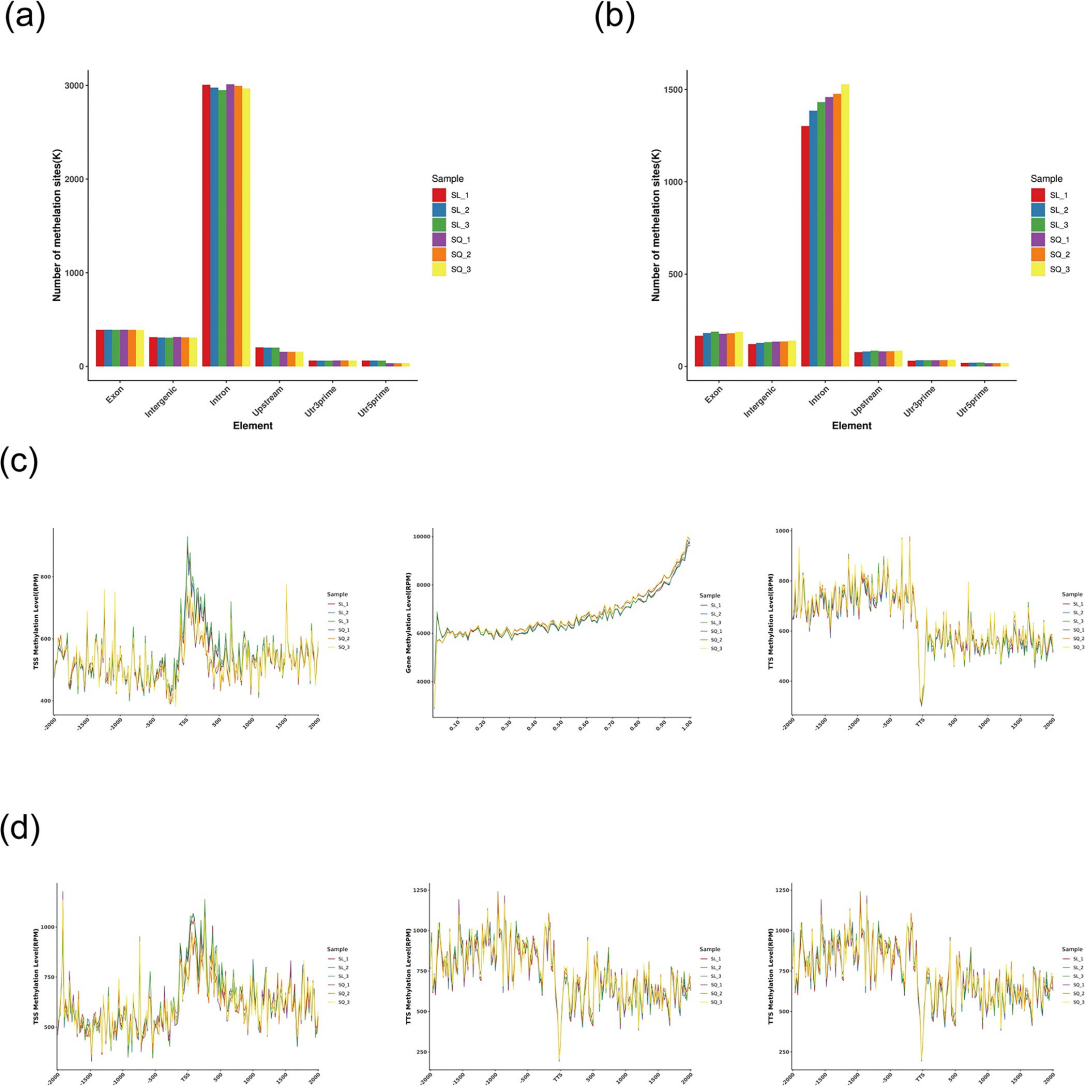
Distribution of DNA methylation sites in different functional regions. (a) Histogram showing the distribution of CCGG-based DMGs on different functional regions of the genome. Y-axis, methylation site count; X-axis, genome constituents. (b) Histogram showing the distribution of CCWGG-based DMGs on different functional regions of the genome. Y-axis, methylation site count; X-axis, genome constituents. (c) Distribution of CCGG-based DMGs around the gene body. X-axis, adjacent position; Y-axis, normalized read number. (d) Distribution of CCWGG-based DMGs around the gene body.

We analyzed the distribution patterns of methylated CCGG and CCWGG sites across various genomic regions, namely the 5’ untranslated region (UTR), 3’ UTR, upstream regions of genes, exons, introns, and intergenic spaces (**Fig 5A and 5B**). Most of the DNA methylation sites were predominantly localized in intronic regions, followed closely by exons, intergenic spaces, and upstream regions. The proportions of methylated sites were minimal in both the 5’ and 3’ UTRs. We also examined the distribution patterns of the 2-kilobase (kb) region upstream of the transcription start site (TSS), the gene body itself, and the TTS. The regions that flank the TSS are important in regulating gene expression. The DNA methylation levels at CCGG and CCWGG sites in the gene body and the TTS had comparable patterns between the control and ARC groups (**Fig 5C and 5D**). The DNA methylation levels were notably lower in the 2-kb region upstream of the TSS and markedly higher at the TSS. Methylation levels gradually increased throughout the gene bodies, and decreased toward the TTS. The distribution of DNA methylation at CCGG sites mirrored the TSS methylation pattern, whereas Contrastingly, the distribution of methylation at CCWGG sites had a distinct pattern. Specifically, methylation levels at CCWGG sites were initially lower in the 2-kb upstream region of the TSS, then abruptly increased proximal to the TSS (left panel, **Fig 5C**). Three pronounced peaks of elevated DNA methylation were detected at CCWGG sites (left panel, **Fig 5D**).

### 5. MethylRAD-seq combined with transcriptome sequencing

**Fig 6:**
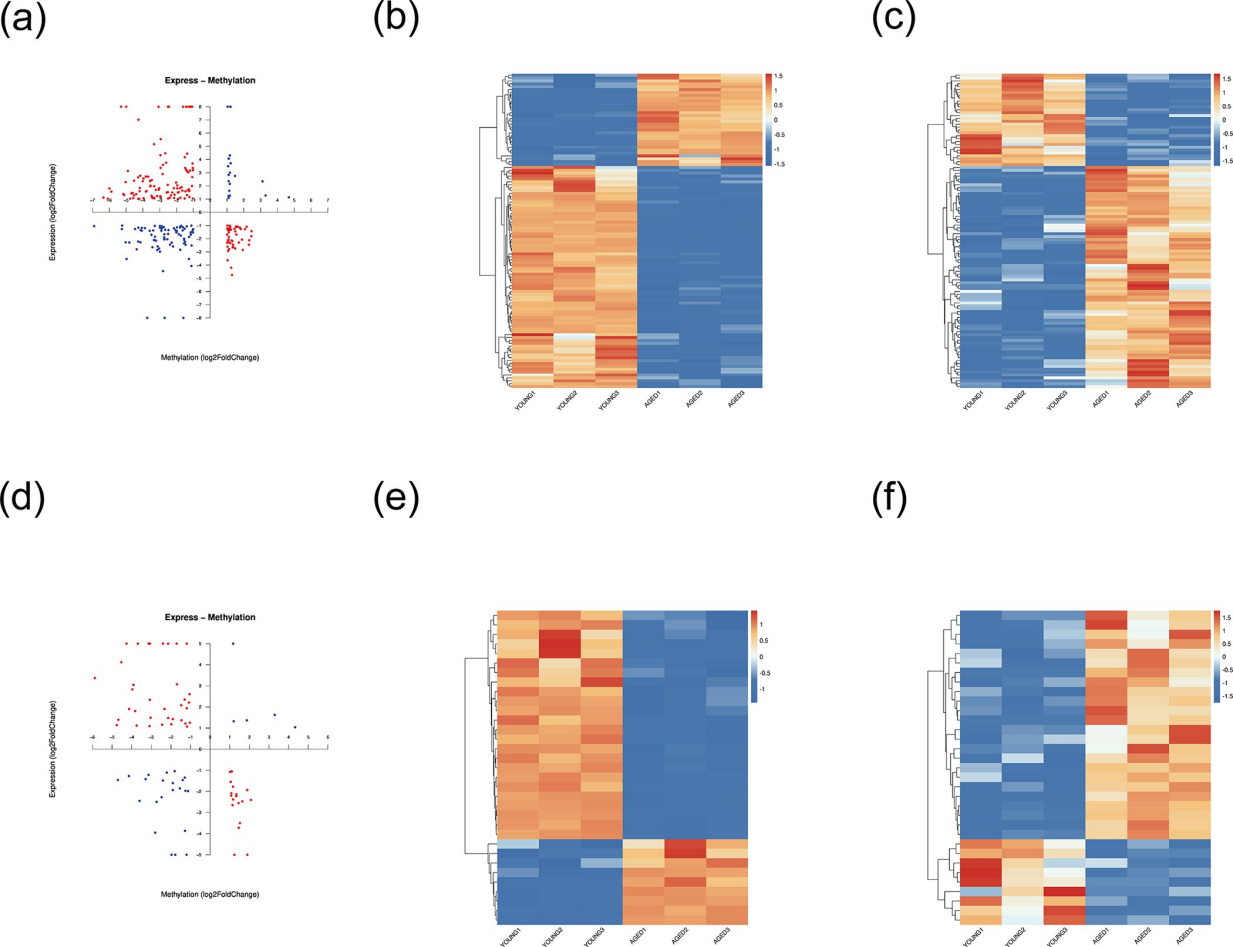
Results of combined analysis of DMGs at DMSs in the promoter region and DEGs. **(**a) A four-quadrant diagram for differentially expressed genes and genes with differential methylation levels at CCGG sites within the promoter region. Red represents negatively correlated sites where the FC of differentially expressed RNA is greater than 2; blue denotes positively correlated sites with FC > 2.The genes in the first quadrant (upper right, blue dots) represent those with both up-regulated gene expression and methylation levels; the genes in the second quadrant (upper left, red dots) indicate those with down-regulated gene expression but up-regulated methylation levels; the genes in the third quadrant (lower left, blue dots) are those with both down-regulated gene expression and methylation levels; the genes in the fourth quadrant (lower right, red dots) contain those with up-regulated gene expression but down-regulated methylation levels. (b) Heat map for hierarchical clustering analysis of differentially expressed genes in the promoter region. On the horizontal axis, each column represents a sample; on the vertical axis, each row represents a gene. The gene expression levels are mapped to the color scale, with red representing high expression and blue representing low expression. On the left is the dendrogram of the cluster analysis of the gene expression patterns.(c) Genes with differential methylation at distinct CCGG methylation sites in the promoter region. On the horizontal axis, each column represents a sample; on the vertical axis, each row represents a gene. The gene methylation levels are mapped to the color scale, with red representing high methylation and blue representing low methylation. On the left is the dendrogram of the cluster analysis of the gene methylation patterns, which shows the similarities between genes.(d) A four-quadrant plot for differentially expressed genes and genes with differential methylation levels at CCWGG sites within the promoter region. Red represents negatively correlated sites where the FC of differentially expressed RNA is greater than 2; blue denotes positively correlated sites with FC > 2. (e) Heat map for hierarchical clustering analysis of differentially expressed genes in the promoter region. Red indicates up-regulation, and blue indicates down-regulation. (f) Genes with differential methylation at diverse CCWGG methylation sites in the promoter region. Blue indicates hypomethylation, and red signifies hypermethylation.DEGs with differential methylation at CCGG/CCWGG sites in the promoter region can be found in S3 and S4 Tables. The Protein-Protein Interaction (PPI) graph of these DEGs with differential methylation at CCGG/CCWGG sites in the promoter region can be found in S1 and S2 Figs, and the corresponding protein interaction scores can be found in S5 and S6 Tables.

We identified 3,856 DEGs between the lens tissues from aged and young mice (*P*-value threshold <0.05, fold change >2.n compared the young group) by analyzing the previously published transcriptome sequencing data from the identical disease model. Among the identified DEGs, 917 were up-regulated and 2,939 were down-regulated, as reported previously [9]. DNA methylation has a pivotal role in regulating gene expression by modulating chromatin architecture, DNA configuration, stability, and protein–DNA interactions. Traditionally, methylation in promoter regions is considered to have a profound effect on gene expression regulation [20], prompting our focus on methylation differences specifically in these regions. Consequently, we integrated the RNA-seq transcriptome data [9] with an analysis of the DNA methylation patterns in promoter regions. To visualize the correlation between DNA methylation and gene expression levels, we constructed four-quadrant plots for CCGG and CCWGG methylation sites (**Fig 6A and 6D**). Red indicates the negatively correlated sites where the FC of differentially expressed RNAs is greater than 2; blue indicates the positively correlated sites where FC > 2. The first quadrant (upper right, blue dots) represents the genes with both upregulated gene expression and methylation levels, which may synergistically promote related biological processes. The second quadrant (upper left, red dots) represents the genes with downregulated gene expression but upregulated methylation levels, suggesting that there may be a mechanism where methylation inhibits expression. The third quadrant (lower left, blue dots) represents the genes with both downregulated gene expression and methylation levels, showing a consistent trend of change. The fourth quadrant (lower right, red dots) contains the genes with upregulated gene expression but downregulated methylation levels, and demethylation may promote their expression.

We filtered out 109 and 33 DEGs that had differential methylation at CCGG and CCWGG sites (S3 **and** S4 **Tables**), respectively, in their promoter regions by applying thresholds of *P*-value <0.05 and correlation coefficient <−0.8. The DEGs that have CCGG sites in their promoter regions are displayed in **Fig 6B**. On the abscissa, each column represents a sample; on the ordinate, each row represents a gene. The gene expression levels are compared against the color scale, with red representing high expression and blue representing low expression. On the left is the dendrogram of the cluster analysis of the gene expression patterns, which shows the similarities between genes. The shorter the branch is, the more similar the expressions of the genes are in different samples.The genes that have differentially methylated CCGG sites in their promoter regions are shown in **Fig 6C**.On the abscissa, each column represents a sample; on the ordinate, each row represents a gene. The gene methylation levels are compared against the color scale, with red representing high methylation and blue representing low methylation. On the left is the dendrogram of the cluster analysis of the gene methylation patterns, which shows the similarities between genes. The shorter the branch is, the more similar the methylation states of the genes are in different samples. The DEGs that have CCWGG sites in their promoter regions are shown in **Fig 6E** (red indicates up-regulation, blue indicates down-regulation). The genes that have differentially methylated CCWGG sites in their promoter regions are presented in **Fig 6F** (blue indicates hypomethylation, red indicates hypermethylation). These results indicate an inverse relationship between gene expression and DNA methylation levels, suggesting that they display opposing trends.

### 6. GO and KEGG enrichment analysis for DEGs with differentially methylated CCGG and CCWGG sites

**Fig 7:**
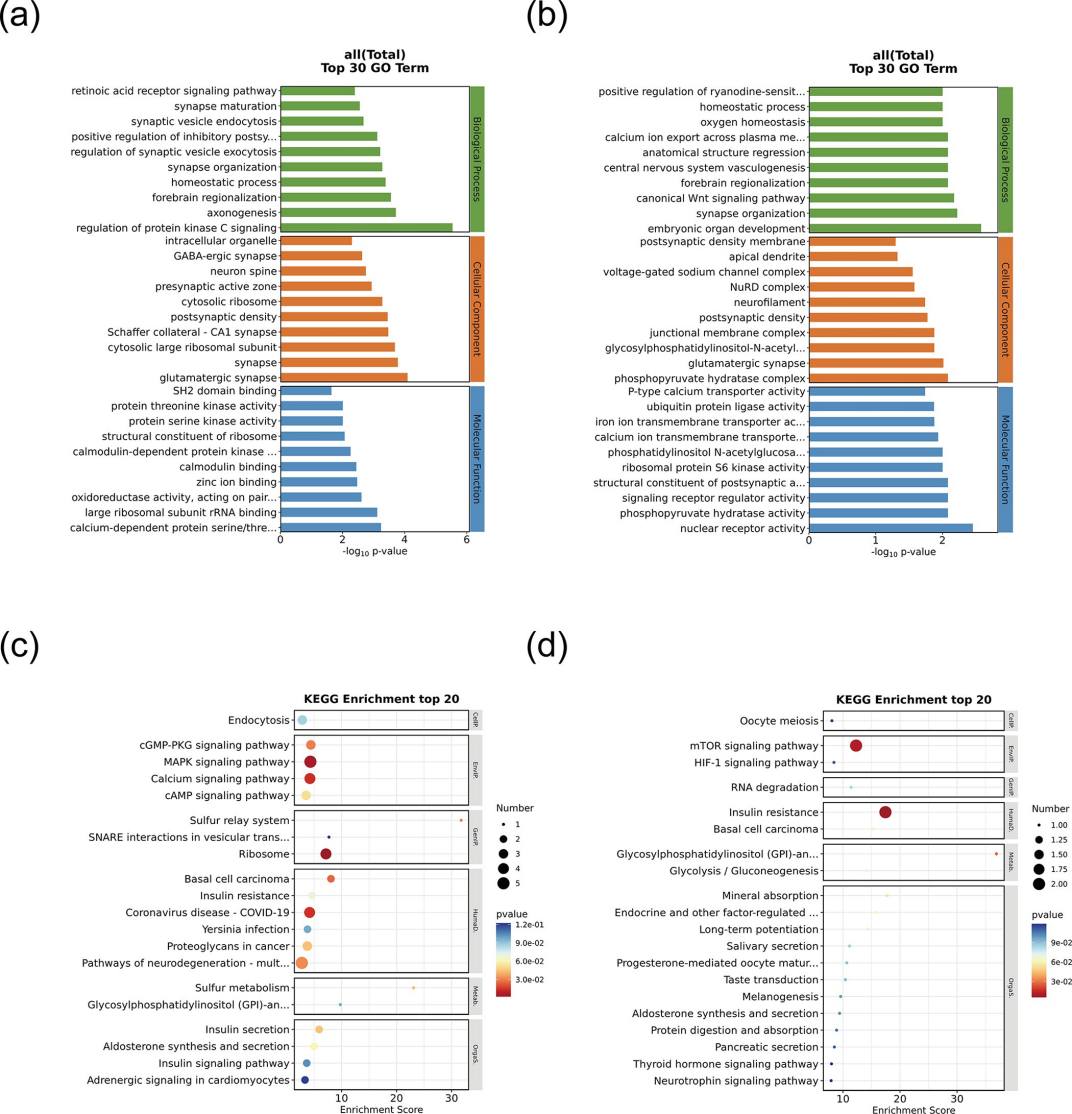
Perform GO and KEGG enrichment analysis for DEGs with differentially methylated levels at CCGG and CCWGG sites. GO annotation shows DMGs related to distinct molecular functions (MF, red), cellular components (CC, blue), and biological processes (BP, green). (a) GO enrichment analysis of DEGs with differentially methylated CCGG sites in promoter region; (b) GO enrichment analysis of DEGs with differentially methylated CCWGG sites in promoter region; (c) KEGG enrichment analysis of DEGs with differentially methylated CCGG sites in promoter region; (d) KEGG enrichment analysis of DEGs with differentially methylated CCWGG sites in promoter region.Significant results of GO enrichment analysis of DEGs with differentially methylated CCGG/CCWGG sites in promoter region (*P* < 0.05, correlation coefficient < -0.8) in **S7** and S8 Tables. Significant results of KEGG enrichment analysis of DEGs with differentially methylated CCGG/CCWGG sites in promoter region (*P* < 0.05, correlation coefficient < -0.8) in S8 and **S10** Tables.

An integrated the analyses of the promoter regions of the DMGs and DEGs identified 109 and 33 genes at CCGG and CCWGG sites, respectively, using a P-value threshold of <0.05 and correlation coefficient of <−0.8, then GO and KEGG functional enrichment analyses were performed on the identified genes (**S7–S10 Tables)**. Among the top 30 GO terms for CCGG-based DMGs, regulation of protein kinase C signaling was highly enriched under biological process (e.g., *Akap12*, *Capzb*), and calmodulin binding (e.g., *Mapkapk3*, *Mknk2*), calmodulin-dependent protein kinase activity (e.g., *Mapkapk3*, *Mknk2*), protein threonine kinase activity (e.g., *Dmpk*, *Mapkapk3*, *Rps6ka3*, *Mknk2*), and SH2 domain binding (*Sqstm1*, *Syp*) were highly enriched under molecular function (**Fig 7A** and **Table 4**). For CCWGG-based DMGs, canonical Wnt signaling pathway (e.g., *Med12*, *Wnt7b*) and homeostatic process (specifically *Wnt7b*) were highly enriched under biological process, and nuclear receptor activity (*Srebf1*, *Esrrg*) and ribosomal protein S6 kinase activity (*Rps6ka3*) were highly enriched under molecular function (**Fig 7B** and **Table 5)**. To predict the pathways involving DEGs with differentially methylated CCGG and CCWGG sites in their promoter regions, we performed KEGG enrichment analysis (**S9 and S10 Tables**). The CCGG-based DMGs were primarily involved in MAPK signaling pathway (e.g., *Mapkapk3*, *Mknk2*), cAMP signaling pathway (e.g., *Cacna1f*, *Atp2b3*), and calcium signaling pathway (e.g., *Slc25a4*, *Cacna1f*) as shown in **Fig 7C**. The CCWGG-based DMGs were primarily involved in the mTOR signaling pathway (e.g., *Rps6k*, *Wnt7b*) and the HIF-1 signaling pathway (including *Eno4*) as shown in **Fig 7C** and **Table 6**. A combined analysis of the GO and KEGG terms and pathways showed that, at CCGG sites, the genes were predominantly associated with regulation of protein kinase C signaling (e.g., *Akap12*, *Capzb*), protein threonine kinase activity (e.g., *Dmpk*, *Mapkapk3*), and the calcium signaling pathway (e.g., *Slc25a4*, *Cacna1f*), whereas, at CCWGG sites, they were associated with ribosomal protein S6 kinase activity (*Rps6ka3*) (**Fig 7D** and **Table 7**).

**Table 4:** The top 30 GO enrichment analysis was conducted on DEGs with differentially methylated levels at CCGG sites in the promoter region.

Category	Term	geneID
biological process	regulation of protein kinase C signaling	Akap12, Capzb, Sez6l2
biological process	axonogenesis	Als2, Atl1, Cacna1f, Nrn1, Slitrk1
biological process	forebrain regionalization	Lhx1, Wnt7b
biological process	homeostatic process	Slitrk1, Wnt7b
biological process	synapse organization	Nlgn3, Prrt1, Syn1, Wnt7b
biological process	regulation of synaptic vesicle exocytosis	Chrnb3, Nrn1, Rims2, Syp
biological process	positive regulation of inhibitory postsynaptic potential	Nlgn3, Rims2
biological process	synaptic vesicle endocytosis	Nlgn3, Synj1, Syp
biological process	synapse maturation	Palm, Sez6l2
biological process	retinoic acid receptor signaling pathway	Cyp26a1, Esrrg
cellular component	glutamatergic synapse	Arpc5l, Atp2b3, Gripap1, Il1rapl2, Nlgn3, Nrn1, Prrt1, Rims2, Slc1a7, Slitrk1, Synj1
cellular component	synapse	Arpc5l, Chrnb3, Gripap1, Mpst, Nlgn3, Nrn1, Palm, Prrt1, Rims2, Rplp0, Slitrk1, Syn1, Syp
cellular component	cytosolic large ribosomal subunit	Rpl12, Rpl13, Rpl14, Rplp0
cellular component	Schaffer collateral—CA1 synapse	Akap12, Capzb, Syn1, Synj1, Syp
cellular component	postsynaptic density	Als2, Capzb, Palm, Rpl12, Rpl14, Rplp0, Syn1, Syp
cellular component	cytosolic ribosome	Rpl12, Rpl13, Rpl14, Rplp0
cellular component	presynaptic active zone	Rims2, Syn1, Syp
cellular component	neuron spine	Palm, Syp
cellular component	GABA-ergic synapse	Atp2b3, Nlgn3, Rims2, Slitrk1
cellular component	intracellular organelle	Syn1, Syp
molecular function	calcium-dependent protein serine/threonine kinase activity	Mapkapk3, Mknk2
molecular function	large ribosomal subunit rRNA binding	Rpl12, Rplp0
molecular function	oxidoreductase activity, acting on paired donors, with incorporation or reduction of molecular oxygen, NAD(P)H as one donor, and incorporation of one atom of oxygen	Cyp26a1, Mical2
molecular function	zinc ion binding	Car10, Esrrg, Hhip, Kdm4c, Lin28a, Pnma3, Siva1, Sqstm1, Zcchc3, Zfand4
molecular function	calmodulin binding	Akap12, Esrrg, Mapkapk3, Mknk2, Myh3
molecular function	calmodulin-dependent protein kinase activity	Mapkapk3, Mknk2
molecular function	structural constituent of ribosome	Rpl12, Rpl13, Rpl14, Rplp0
molecular function	protein serine kinase activity	Cilk1, Dmpk, Mapkapk3, Mknk2, Rps6ka3
molecular function	protein threonine kinase activity	Cilk1, Dmpk, Mapkapk3, Mknk2, Rps6ka3
molecular function	SH2 domain binding	Sqstm1, Syp

**Table 5:** The top 30 GO enrichment analyses for DEGs that have differentially methylated levels at CCWGG sites within the promoter region.

Category	Term	geneID
biological process	embryonic organ development	Med12, Wnt7b
biological process	synapse organization	Prrt1, Wnt7b
biological process	canonical Wnt signaling pathway	Med12, Wnt7b
biological process	forebrain regionalization	Wnt7b
biological process	central nervous system vasculogenesis	Wnt7b
biological process	anatomical structure regression	Wnt7b
biological process	calcium ion export across plasma membrane	Atp2b3
biological process	oxygen homeostasis	Wnt7b
biological process	homeostatic process	Wnt7b
biological process	positive regulation of ryanodine-sensitive calcium-release channel activity	Jph2
cellular component	phosphopyruvate hydratase complex	Eno4
cellular component	glutamatergic synapse	Atp2b3, Il1rapl2, Ina, Prrt1
cellular component	glycosylphosphatidylinositol-N-acetylglucosaminyltransferase (GPI-GnT) complex	Piga
cellular component	junctional membrane complex	Jph2
cellular component	postsynaptic density	Neurl1a, Rpl14, Srcin1
cellular component	neurofilament	Ina
cellular component	NuRD complex	Mta3
cellular component	voltage-gated sodium channel complex	Scn4a
cellular component	apical dendrite	Neurl1a
cellular component	postsynaptic density membrane	Prrt1
molecular function	nuclear receptor activity	Esrrg, Srebf1
molecular function	phosphopyruvate hydratase activity	Eno4
molecular function	signaling receptor regulator activity	Prrt1
molecular function	structural constituent of postsynaptic actin cytoskeleton	Ina
molecular function	ribosomal protein S6 kinase activity	Rps6ka3
molecular function	phosphatidylinositol N-acetylglucosaminyltransferase activity	Piga
molecular function	calcium ion transmembrane transporter activity	Atp2b3
molecular function	iron ion transmembrane transporter activity	Slc25a28
molecular function	ubiquitin protein ligase activity	Med12, Neurl1a, Rnf122
molecular function	P-type calcium transporter activity	Atp2b3

**Table 6:** Top 20 KEGG enrichment analyses are done for DEGs with varying methylation levels at CCGG sites in the promoter region.

Term	geneID
Ribosome	Rpl12, Rpl13, Rpl14, Rplp0
MAPK signaling pathway	Cacna1f, Mapkapk3, Mknk2, Pdgfra, Rps6ka3
Calcium signaling pathway	Atp2b3, Cacna1f, Pdgfra, Slc25a4
Coronavirus disease—COVID-19	Rpl12, Rpl13, Rpl14, Rplp0
Basal cell carcinoma	Hhip, Wnt7b
cGMP-PKG signaling pathway	Atp2b3, Cacna1f, Slc25a4
Sulfur relay system	Mpst
Pathways of neurodegeneration—multiple diseases	Als2, Cacna1f, Slc25a4, Sqstm1, Wnt7b
Sulfur metabolism	Mpst
Proteoglycans in cancer	Ank1, Hspb2, Wnt7b
Insulin secretion	Cacna1f, Rims2
cAMP signaling pathway	Atp2b3, Cacna1f, Hhip
Aldosterone synthesis and secretion	Atp2b3, Cacna1f
Insulin resistance	Rps6ka3, Srebf1
Endocytosis	Arpc5l, Capzb, Pdgfra
Yersinia infection	Arpc5l, Rps6ka3
Glycosylphosphatidylinositol (GPI)-anchor biosynthesis	Piga
Insulin signaling pathway	Mknk2, Srebf1
Adrenergic signaling in cardiomyocytes	Atp2b3, Cacna1f
SNARE interactions in vesicular transport	Use1

**Table 7:** Top 20 KEGG enrichment analyses for DEGs with different methylation levels at CCWGG sites in the promoter region.

Term	geneID
Insulin resistance	Rps6ka3, Srebf1
mTOR signaling pathway	Rps6ka3, Wnt7b
Glycosylphosphatidylinositol (GPI)-anchor biosynthesis	Piga
Mineral absorption	Atp2b3
Endocrine and other factor-regulated calcium reabsorption	Il1rapl2
Basal cell carcinoma	Wnt7b
Long-term potentiation	Rps6ka3
Glycolysis / Gluconeogenesis	Eno4
RNA degradation	Eno4
Salivary secretion	Atp2b3
Progesterone-mediated oocyte maturation	Rps6ka3
Taste transduction	Kcnk5
Melanogenesis	Wnt7b
Aldosterone synthesis and secretion	Atp2b3
Protein digestion and absorption	Kcnk5
Pancreatic secretion	Atp2b3
HIF-1 signaling pathway	Eno4
Oocyte meiosis	Rps6ka3
Thyroid hormone signaling pathway	Med12
Neurotrophin signaling pathway	Rps6ka3

### 7. Pyrophosphate sequencing validation

**Fig 8:**
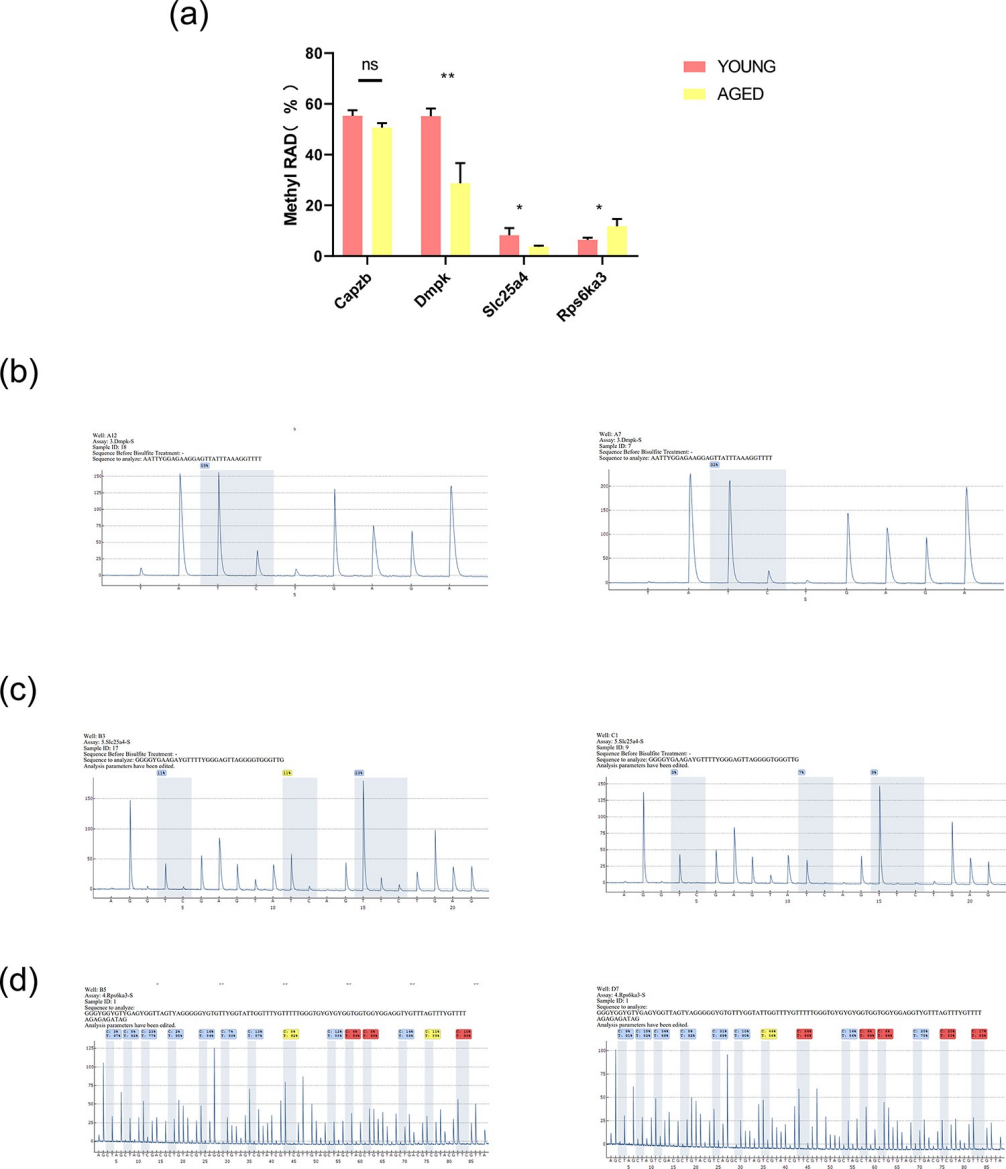
Measurement of gene methylation using pyrophosphate sequencing. (a) The bar charts showing the statistical results of the methylation levels of *Dmpk*, *Slc25a4* and *Rps6ka3* in the two groups of samples are presented. ("ns" indicates no statistical difference;*indicates *P* less than 0.05; ** indicates *P* less than 0.01.) (b-d) The representative results of pyrophosphate sequencing for the Young and Aged groups are presented.(b) The pyrosequencing peak plots of the methylation sites where the *Dmpk* gene is located in the young and aged mouse groups. The left figure represents the methylation level of the methylation site where the *Dmpk* gene is located in the young mouse group, and the right figure represents the methylation level of the methylation site where the *Dmpk* gene is located in the aged mouse group. The horizontal axis shows the order of added dNTPs, and the vertical axis represents the intensity of the fluorescent signal (the detection signal of the methylated fragment).(c) The pyrosequencing peak plots of the methylation sites where the *Slc25a4* gene is located in the young and aged mouse groups. The height of the peaks reflects the signal intensity of the methylation sites. The higher the intensity, the higher the degree of methylation of the site in the sample.(d) The pyrosequencing peak plots of the methylation sites where the *Rps6ka3* gene is located in the young and aged mouse groups. The sequencing quality corresponding to blue is the best, followed by yellow, and red indicates poor quality. If blue appears in a certain reaction, it means that the overall quality is within an acceptable range and there are no significant problems. The specific methylation site where the gene is located is marked by the gray shaded area.

To evaluate the reliability and precision of the MethylRAD-seq data, we validated four genes (*Capzb*, *Dmpk*, *Slc25a4*, *Rps6ka3*) by pyrophosphate sequencing. These genes were selected based on the comprehensive analysis of methylation and transcriptome (**Fig 7A**). We performed pyrosequencing verification on five samples from the young group (normal group), that is, n = 5, named Young, and five samples from the naturally aging group (ARC group), named Aged. We found that the methylation status of these genes generally concurred with the sequencing outcomes obtained from the MethylRAD, confirming the reliability of the initial findings. Specifically speaking:the methylation levels of *Dmpk* (**Fig 8B**) and Slc25a4 (**Fig 8C**) were lower in the ARC group than they were in the control group, whereas the methylation level of Rps6ka3 (**Fig 8D**) was higher in the ARC group than it was in the control group (The average methylation rate of *Dmpk* was 59% in the young mouse group and 32% in the aged mouse group, with a *p*-value of less than 0.01, indicating significant statistical significance. The average methylation rate of *Slc25a4* was 15% in the young mouse group and 6% in the aged mouse group, with a p-value of less than 0.05, showing statistical significance. The average methylation rate of *Rps6ka3* was 13.5% in the young mouse group and 18% in the aged mouse group, with a *p*-value of less than 0.05, also demonstrating statistical significance). These findings align with our integrated analysis. No notable distinction was detected in methylation level of Capzb between the two groups, prompting a further analysis of the remaining three genes.

## Discussion

Approximately 94 million individuals worldwide are blind or visually impaired, and cataract is the most prevalent cause of blindness [21]. Although cataract surgery is an increasingly prevalent treatment, high costs and postoperative complications still affect many patients. Data show that if the occurrence of ARC can be postponed by a decade, the number of surgeries will decrease by half [22]. Aging has become a major risk factor for various human diseases, including diabetes, cancer, cardiovascular diseases, and neurodegenerative diseases [23–25]. Among them, the onset of ARC is also closely related to age [26]. Currently, no breakthrough in drug treatment for ARC is currently available, and the unclear mechanism of lens aging is the major limiting bottleneck. In epigenetics research, numerous aspects need to be considered, including DNA methylation, histone modification, and microRNAs [27]. Previous studies have suggested that DNA methylation modifications influenced by both age and the surrounding environment, likely play a pivotal role in the pathogenesis of cataracts and the developmental processes of the lens [28]. Aberrant methylation, as a fundamental aspect of epigenetics, is one of the key factors leading to eye diseases, especially cataracts. Notably, in senile cataracts, differential epigenetic patterns in DNA surrounding specific genes are found. Studies on cataract models have detected age-dependent methylation increase in the Klotho gene promoter [29]. At the same time, lens crystallins play a crucial role in maintaining lens transparency. Among them, α-crystallin, as the main structural protein component, accounts for 35% of all crystallins in the lens and acts as a molecular chaperone to prevent the aggregation of other crystallins [30,31]. In age-related nuclear cataracts, the level of α-crystallin is reduced, which is related to the hypermethylation of CpG islands in the *CRYAA* gene promoter. Treatment with DNA methylation inhibitors can restore *CRYAA* gene expression [32]. In addition, there is epigenetic silencing of nuclear factor erythroid 2-related factor 2 (*Nrf2*). *Nrf2* is a transcriptional activator that can protect the lens but is negatively correlated with the *Keap1* protein. The expression of *Keap1* increases with age and can stimulate proteasome-mediated degradation of *Nrf2* and inhibit the *Nrf2*-dependent antioxidant protection of the lens. Analysis of age-related cataract lenses shows significant demethylation of *Keap1* and a decrease in *Nrf2* [33]. These studies provide strong evidence for the importance of epigenetics in cataracts and bring new hope for the development of targeted treatment methods for cataracts.However, previous studies on the methylation of pathogenic genes for ARC focus on one site, multiple sites, or a single gene, while the research on genome-wide methylation is relatively lacking. Therefore, this article has significant advantages in conducting genome-wide methylation research on cataracts. Genome-wide methylation research can comprehensively and systematically discover epigenetic changes related to the occurrence and development of cataracts, which helps to understand its pathogenic mechanism and provides potential targets for precise treatment.

For ophthalmic diseases, DMSs also have diagnostic value. A comprehensive study identified significant variations in mitochondrial DNA methylation in the peripheral blood of patients with diabetes, differentiating patients with retinopathy from those without [34–36]. It has also been shown that the hypomethylated state of the promoters of *NLRP3*, *TGFβ1*, *MCP-1*, and *TNFSF2* may increase the risk of developing diabetic retinopathy [37]. This observation result shows that the methylation patterns of these genes have the potential to be biomarkers for early diagnosis and are of great value. At the same time, they can also provide key insights for targeted therapy. Drug intervention treatment for abnormal methylation sites can open up broad prospects for non-surgical treatment of ARC.

Genome-wide methylation studies can comprehensively and systematically discover epigenetic alterations associated with the occurrence and progression of cataracts, which assists in understanding the pathogenic mechanism and offers potential targets for precise treatment. Hence, in-depth exploration of the mechanism by which DNA methylation affects the pathogenesis of ARC is of great significance. Changes in methylation patterns are expected to indicate potential therapeutic targets. Regulating methylation levels can improve gene expression and open up new avenues for drug treatment. In addition, DMG sites have early diagnostic significance and can improve diagnostic accuracy and identify high-risk populations. In this study, we used MethylRAD-seq technology to detect genome-wide methylation and conducted a combined genome-wide analysis of methylation with transcriptomic analysis to expand the research scope of ARC, contribute to new perspectives and achievements to this field, and promote new breakthroughs in cataract research and treatment.

Numerous animal model studies have discovered 180 cataract-related genes in mice [38,39]. Ultraviolet induction or selenite induction methods have been used to construct mouse cataract models. Although models can be obtained in a short time, it is difficult to truly simulate the natural aging process of the lens [40–43]. Therefore, in this study, we constructed a naturally aging mouse model that simulates the disease process of human ARC more accurately and truly than previous models. Our model reflects the dynamic changes of the natural aging of the lens with time, thereby providing a more reliable reference for studying the pathogenesis of human cataracts. This mouse model was successfully verified in our previous research to be capable of simulating the progression of human ARC. Importantly, this model fills the deficiency in the study of DNA methylation sequencing in animal models of cataract and offers a new tool for a profound exploration of the genetic and epigenetic mechanisms of cataracts. By conducting DNA methylation analysis of the anterior lens capsule of naturally aging mice, alterations in the regulation of ARC-related gene expression were discovered. This provides a foundation for the early diagnosis and intervention of ARC.

In this study, we conducted MethylRAD-seq analysis on the anterior lens capsules of young and aged mouse cohorts and identified 76,524 and 15,608 DMSs at CCGG and CCWGG sites, respectively (**S1 and S2 Tables**). The comprehensive GO and KEGG functional enrichment analyses indicated the functional significance of the DMGs located at these sites. The top 30 GO terms for overall DMGs at CCGG sites were mainly related to the activity of store-operated calcium channels (*Trpc7*, *Trpc1*, *Trpm8*) (**Fig 4A**). The emergence of cataracts is strongly associated with calcium pumps. Strict regulation of Ca^2+^ influx into LECs is crucial because the calcium concentration has profound effects on maintaining lens homeostasis and growth.When there is an intracellular overload of Ca^2+^, adverse events are triggered. Properly maintaining the Ca^2+^ level can prevent cataracts [44], which indicates the accuracy of the GO analysis of overall DMGs at CCGG sites related to calcium channel activity.

The top 30 GO terms for overall DMGs at CCWGG sites were mainly related to cellular sodium homeostasis (*Atp1b3*, *Slc8a3*, *Atp1b1*) (**Fig 4B**). Previous studies have confirmed the close relationship between cataracts and sodium pumps [45]. Oxidative stress caused by disparity between oxidizing agents and antioxidants and disturbance of redox signaling can damage tissues and cells [46]. Oxygen free radicals are involved in lens aging [47]. Antioxidant enzymes can prevent the deterioration of Na^(+)^-K^(+)^-ATPase-dependent pumps [45]. Deterioration of sodium pumps is important in cataract development, confirming the accuracy of the sequencing result.

The top 20 primary KEGG pathways identified at CCGG sites were predominantly linked to the MAPK signaling cascade and calcium signaling pathway, featuring genes such as *Mapk1*, *Mapk3*, *Fgf9*, *and Slc25a4* (**Fig 4C**). MAPKs have been classified into four subfamilies, namely *ERK1/2*, *JNK1/2/3*, *P38*, and *ERK5*. MAPKs participate in cell differentiation, proliferation, and apoptosis processes. Lens epithelial cell apoptosis, a prevalent mechanism underlying cataract development, is triggered by oxidative stress. Suppression of phosphorylation of *ERK1/2*, *JNK*, and *P38* shows the relationship between the MAPK pathway and cataracts [48]. Additionally, intracellular Ca^2+^ imbalance causes LEC death and leads to cataracts. The top 20 KEGG pathways from the sequencing analysis are consistent with these findings.

Studies of *Rap1* in ophthalmic diseases have focused on lens epithelium [49], and retina [50] and cornea [51]. Currently, no studies have directly linked *Rap1* and ARC. Our sequencing results show Rap1 signaling pathway is in top 20 of KEGG pathways enriched by overall DMGs at CCWGG sites (**Fig 4D**), suggesting a possible connection with ARC and providing new clues. The Hippo signaling pathway was one of the top 20 KEGG pathways for overall DMGs at CCWGG sites (**Fig 4D**), featuring genes such as *Mapk1*, *Mapk3*, *Yap1*. Importantly, in our previous study, we confirmed that *YAP1* and *MST2* in the Hippo signaling pathway regulated LEC apoptosis [52] related to cataract occurrence. This finding also reflects the accuracy of the sequencing analysis.

It is known that hypermethylation in the promoter region usually leads to transcriptional repression, while hypomethylation may result in increased gene expression [53–56]. The research by Balázs Gyȍrffy et al., on the other hand, indicates that under the drive of methylation, the phenomenon of decreased *SFRP1* expression can be used to predict the poor prognosis of patients with ER1/HER22 breast cancer (BCs) [57].Therefore, in this paper, we focused on the study of promoter methylation to screen genes.We combined transcriptome sequencing and MethylRAD-seq results to select common DEGs. A total of 109 DEGs with negative methylation at CCGG sites and 33 DEGs with negative methylation at CCWGG sites were identified by Pearson correlation analysis (S3 and S4 **Tables**). Moreover, the PPI graph of these DEGs with differential methylation at CCGG/CCWGG sites in the promoter region can be found in **S1** and **S2 Figs.**, and the corresponding protein interaction scores can be found in S5 and S6 **Tables**. A joint analysis of DMGs and DEGs at CCGG sites was followed by GO enrichment analysis. The primary enriched term under biological process was modulation of protein kinase C signaling, specifically involving *Capzb*. In this sequencing result, when comparing the elderly group of mice with the young group of mice, it was found that *Capzb* in the promoter region of the elderly group of mice showed a hypomethylated state and its expression level is significantly increased (Highly expressed *Capzb* regulates the occurrence of apoptosis by modulating Protein Kinase C signaling.) (S3 **Table**). This result leads to a high correlation between MAPKAPK and ARC, suggesting that it may play an important role in the formation of ARC.Under the aspect of molecular function, the enriched terms were mainly related to calmodulin binding (*Mapkapk3*, *Mknk2*) and protein serine kinase activity (*Dmpk*, *Rps6ka3*). (**Fig 7A**). Meanwhile, in this sequencing result, compared with young mice, *Rps6ka3* in the promoter region of elderly mice shows hypermethylation and low expression; while *Mapkapk3*, *Mknk2*, and *Dmpk* are hypomethylated and highly expressed (S3 **Table**) (The highly expressed *Mapkapk3* regulates the occurrence of cell apoptosis by modulating the cascade reaction of the MAPK pathway).This result shows a high correlation between *Mapkapk3* and ARC. In addition,according to relevant literature reports, during the physiological activities of the lens, there are some factors that are crucial for maintaining the stability and transparency of the lens. Calmodulin and calcium ions are the key regulatory factors among them. The balance between the two is of vital importance to the health of the lens. Once this balance is disrupted, cataract [58] will be induced. This research result strongly confirms the accuracy of our sequencing results and also fully demonstrates the important status of calmodulin and calcium ions in the pathogenesis of cataract.

A joint analysis of DMGs and DEGs at CCWGG sites in the promoter region was followed by GO enrichment analysis. The primary enriched term was canonical Wnt signaling pathway (**Fig 7B**), featuring genes such as *Med12* and *Wnt7b*. Meanwhile, in this sequencing result, compared with young mice, *Wnt7b* in the promoter region of elderly mice shows hypomethylation and high expression; while *Med12* is hypermethylated and lowly expressed (S4 **Table**). As shown in the literature,some human eye disorders are triggered by mutations of genes in the Wnt signaling pathway, including cataracts, retinal degeneration, and ocular tumors [59]. These diseases are also associated with nuclear receptor activity (*Srebf1*). The transition of LECs from an epithelial to mesenchymal phenotype is the central pathological process underlying fibrotic cataracts. Lanosterol synthase can potentially impede this deleterious transformation by modulating *Srebf1* [60]. Previous research was limited to the relationship between congenital cataracts and nuclear receptor activity, whereas, in this study, a connection between *Srebf1* in nuclear receptor activity and ARC has been established,that is, That is, compared with young mice, *Srebf1* in the promoter region of elderly mice (i.e., the ARC group) shows hypomethylation and high expression(S4 **Table**).

A joint analysis of DMGs and DEGs at CCGG and CCWGG sites in the promoter region was followed by KEGG pathway enrichment analysis. The enriched pathways at CCGG sites were mainly related to the MAPK signaling pathway (e.g., *Mapkapk3*, *Mknk2*) and cAMP signaling pathway (*Cacna1f*, *Atp2b3*) (**Table 6**). Meanwhile, in this sequencing result, compared with young mice, *Mknk2* in the promoter region of elderly mice shows hypomethylation and high expression;while *Cacna1f and Atp2b3* are hypermethylated and lowly expressed. (S3 **Table**). And the enriched pathways at CCWGG sites are mainly related to the mTOR signaling pathway (*Rps6ka3*, *Wnt7b*) and HIF-1 signaling pathway (*Eno4*) (**Fig 7C and 7D**). We selected four of the annotated genes from the enrichment analyses as candidate genes (*Dmpk*, *Slc25a4*, *Rps6ka3*, *Capzb*), and verified them by pyrosequencing methylation analysis (**Fig 8A**). In general, the pyrosequencing verification results were consistent with the MethylRAD-seq results. The findings indicate that the methylation levels of *Dmpk* and *Slc25a4* in the anterior lens capsule of naturally aging mice were lower and the methylation level of *Rps6ka3* was higher than their methylation levels in the young mice, which is consistent with the results from the joint analysis. However, no substantial change in the methylation level of *Capzb* was observed between the two groups.

Dystrophia myotonica protein kinase *(Dmpk*) has a kinase domain and a coiled-coil domain that is involved in polymerization. *Dmpk* belongs to the serine/threonine kinase family [61]. *Dmpk* transcripts and protein have been detected in ocular tissues of adults and fetuses. Extension of CTG repeat sequences in the *Dmpk* gene causes type 1 myotonic dystrophy (DM1). Cataract is the main ocular complication of patients with DM1 with unknown mechanism [62]. *P53* was shown to induce *Dmpk* expression by up-regulating *P73* expression and promoting apoptosis [63]. Apoptosis of LECs can also lead to cataract.Then, is this a potential mechanism by which *Dmpk* causes cataract in DM1 patients? This needs further study.

The *Slc25* family, also known as the mitochondrial carrier family, is the most extensive protein transport gene family in the human solute carrier superfamily, with potential involvement in cancer progression [64]. Specifically, the mitochondrial ADP/ATP carrier, also known as adenine nucleotide translocase 1 (*ANT*) or *Slc25a4*, facilitates the import of ADP into the mitochondrial matrix and the export of ATP, which is a crucial step in oxidative phosphorylation [65]. Although *Slc25a4* is known to induce cell death by up-regulating pro-apoptotic protein Bax [66], its relationship with ophthalmic diseases, especially ARC, needs further study. Previous research has demonstrated that *Dmpk* is associated with calcium ions, and the calcium signaling pathway is associated with cataract [67,68]. *Slc25a4* is also related to the calcium signaling pathway [69]. Therefore, we verified *Slc25a4* and *Dmpk* methylation by pyrosequencing. The results were in line with the MethylRAD-seq results. Most of the research on *Rps6ka3* pertains to Coffin-Lowry syndrome [70], and currently, there are no reports of its association with cataracts. However, studies have indicated that *Rps6ka3* may play a crucial role in apoptosis by activating the cAMP response element-binding protein [71]. In this study, the pyrosequencing methylation analysis detected an elevated methylation level of *Rps6ka3* in the anterior lens capsule of aged mice. This finding offers a new orientation for understanding the methylation regulatory role of *Rps6ka3* in ARC. In future studies, we plan to further investigate the mechanism through which these three genes act in the emergence and development of ARC. These genes could potentially serve as therapeutic targets for preventing and intervening in ARC.

To enhance the clinical significance and validate the research results, we compared the differentially expressed genes *Dmpk*, *Slc25a4* and *Rps6ka3* verified by pyrosequencing with the GSE241767 dataset related to human cataract samples in the GEO database (http://www.ncbi.nlm.nih.gov/geo/). Through the comparison, it was found that the differential methylation trends of these genes in human cataracts were consistent with those in our mouse samples.Specifically, in the GSE241767 dataset, compared with the NOR group, the Dmpk in the ARC group showed a hypomethylation state; *Slc25a* was also hypomethylated, while *Rps6ka* was in a hypermethylation state. In our mouse data, compared with the Young group, the *Dmpk* in the Aged group was hypomethylated; *Slc25a4* was hypomethylated and *Rps6ka3* was hypermethylated, and this trend was consistent with the GSE241767 dataset (**S11 and S12 Tables**).By comparing with the human dataset, the clinical significance of the identified genes can be determined more accurately. This measure not only bridges the gap between animal model research and human applications, but also greatly improves the clinical relevance of our research work. At the same time, it provides corresponding clinical support evidence for the effectiveness of these genes as biomarkers of ARC in the future.

Meanwhile, existing literature shows that hematological biomarkers can simplify the search for potential molecular defects in families with congenital cataracts [72]. Additionally, as ARC severity increases, connective tissue growth factor in aqueous humor tends to increase, while ascorbic acid concentration and total antioxidant capacity of aqueous humor are negatively correlated with cataract severity [73]. These results provide ideas for subsequent exploration. Detecting the differential expression of target genes (*Dmpk*, *Slc25a4*, *Rps6ka3*) may offer potential biomarkers for early cataract diagnosis, enabling earlier detection of cataract risk and more timely clinical intervention. Moreover, in—depth study of target genes may provide new targets for cataract treatment. We can conduct drug R & D targeting the signaling pathways of these genes to delay or prevent cataract progression.

In this study, MethylRAD-Seq is used instead of bisulfite sequencing. Although there may be some limitations, such as its detection of methylation sites is relatively incomplete and not as detailed, it is valuable in specific situations.MethylRAD-Seq has high cost-effectiveness and is particularly important in studies involving a large number of clinical samples. It can provide valuable insights into disease mechanism research and potential biomarker discovery at a relatively low cost, as well as information on DNA methylation patterns. It is a useful tool in large-scale analysis studies. In addition, it has unique advantages: simple and fast library construction, strong flexibility to control methylation tag density, good repeatability, small amount of starting DNA suitable for limited samples, no dependence on genome sequences for developing unknown sites and studying differences [74–78]. However, we must also admit that it may have limitations in some specific research needs. In the future, we can consider combining other sequencing methods to more comprehensively understand the patterns and functions of DNA methylation.

Moreover, due to the extremely small anterior lens capsule of mice, only methylation analysis can be performed alone. If the same sample is split, only micro-transcriptome sequencing is possible. But the mouse anterior lens capsule is tiny, leading to insufficient sample size and not meeting the sequencing machine’s minimum requirement. Thus, only conventional transcriptomics on the complete sample is viable. Conventional transcriptomics is better than micro-transcriptomics in data quality (higher sequencing depth/coverage, detecting low-abundance transcripts, accurately quantifying gene expression and providing reliable differential expression results), experimental operation (mature/stable/simple with good repeatability and lower sample requirements), and application scope (suitable for various research needs and easy integration with other omics data for systems biology research). In many previous studies, samples from different batches were also used to carry out multi-omics research work. Xu Zhang et al. employed a two-stage design for methylome and transcriptome study. Stage 1: methylation levels of 99 ankylosing spondylitis (AS) patients and 99 healthy controls (HCs)measured. Stage 2: mRNA levels of 20 patients and 20 HCs measured. Found IL12B gene’s DNA methylation and transcriptome traits can distinguish AS patients from HCs, and hypermethylation may contribute to AS pathogenesis [79]. Yilin Zhao et al. comprehensively analyzed proteome and transcriptome of lens epithelium and fibers. At least 525 proteins in lens can generate info on key lens formation processes and are related to different cells and molecules [80]. However, interpreting methylation data alone often faces many challenges. Therefore, we use pyrosequencing to verify methylation data. This method can accurately detect the degree and site of DNA methylation [81–83]. It not only confirms the correctness of the data but also provides important clues for subsequent clinical verification sites. In clinical practice, these verified methylation sites can serve as potential biomarkers for the diagnosis, prognosis evaluation, and treatment monitoring of ARC disease. Detecting these methylation sites can provide a foundation for the early diagnosis and individualized treatment of ARC. This also provides a theoretical basis for treating ARC by targeting these methylation sites.

In summary, we conducted whole-genome methylation and transcriptome analyses on young and aged mice to identify potential epigenetic biomarkers for ARC. Using this approach, we successfully identified key molecular markers for the occurrence and progression of ARC. These markers may enable early diagnosis and targeted intervention for high-risk populations, and also hold promise for the discovery of new drug targets that can help improve the well-being of patients with ARC. In future studies, in-depth exploration of the different signaling pathways, key regulatory factors, and molecular targets identified in this study can be carried out. The sequencing results can be used to develop clinical diagnostic and treatment tools. Conducting clinical trials to promote the applications of research findings, establishing a long-term monitoring system to track disease progression and treatment effects, and exploring lifestyle intervention measures for preventing ARC will contribute to the provision of personalized prevention recommendations for the public. Overall, this study provides a powerful tool for in-depth understanding of ARC and opens up new frontiers in the prevention, diagnosis, and treatment of ARC.

## Conclusions

DNA methylation is a significant component of epigenetics, and is important in the pathogenesis of cataracts. In this study, we aimed to discover potential epigenetic biomarkers of ARC by integrating whole-genome methylation and transcriptome analyses of young and aged mice. MethylRAD-Seq was performed to examine the whole-genome methylation in the anterior lens capsule tissues of young and aged mice. The methylation analysis identified 76,524 and 15,608 DMSs at CCGG and CCWGG sites. Subsequently, the promoter regions of DMGs were combined with the DEGs obtained by transcriptome sequencing analysis, and 109 and 33 DEGs that were negatively correlated with methylation were identified at CCGG and CCWGG sites, respectively. The combined GO and KEGG annotations identified four ARC-related genes, namely *Capzb*, *Dmpk*, *Slc25a4*, and *Rps6ka3*. These genes were verified by pyrosequencing and the results were consistent with those of MethylRAD-seq. Our investigation provides comprehensive whole-genome DNA methylation patterns and gene expression signatures associated with ARC. The identified methylation-based molecular markers hold promise for future applications in ARC prevention, diagnosis, treatment strategies, and prognostic evaluations.

## Supporting information

S1 Fig The PPI graph of DEGs with differential methylation at CCGG sites in the promoter region. (TIF)

S2 Fig The PPI graph of DEGs with differential methylation at CWGG sites in the promoter region. (TIF)

S1 Table List of all DMSs at CCGG sites. (XLS)

S2 Table List of all DMSs at CCWGG sites. (XLS)

S3 Table List of DEGs with differential methylation at CCGG sites in the promoter region. (XLS)

S4 Table List of DEGs with differential methylation at CCWGG sites in the promoter region. (XLS)

S5 Table The protein interaction scores of DEGs with differential methylation at CCGG sites in the promoter region. (XLS)

S6 Table The protein interaction scores of DEGs with differential methylation at CCWGG sites in the promoter region. (XLS)

S7 Table List of GO enrichment analysis for DEGs with differentially methylated CCGG sites in promoter region. (XLSX)

S8 Table List of GO enrichment analysis for DEGs with differentially methylated CCWGG sites in promoter region. (XLSX)

S9 Table List of KEGG enrichment analysis for DEGs with differentially methylated CCGG sites in promoter region. (XLSX)

S10 Table List of KEGG enrichment analysis for DEGs with differentially methylated CCWGG sites in promoter region. (XLSX)

S11 Table Differential methylation data of human cataracts in the GEO database (GSE241767). (XLSX)

S12 Table The comparison between the methylation data of mice and that of human cataracts (GSE241767). (XLSX)
