# Supplementary figures and images for: Genome-wide DNA methylation and transcriptome sequencing analyses of lens tissue in an age-related mouse cataract model
Source: PLoS One. 2025 Jan 30;20(1):e0316766. doi: 10.1371/journal.pone.0316766 (PMC11781636; doi:10.1371/journal.pone.0316766)

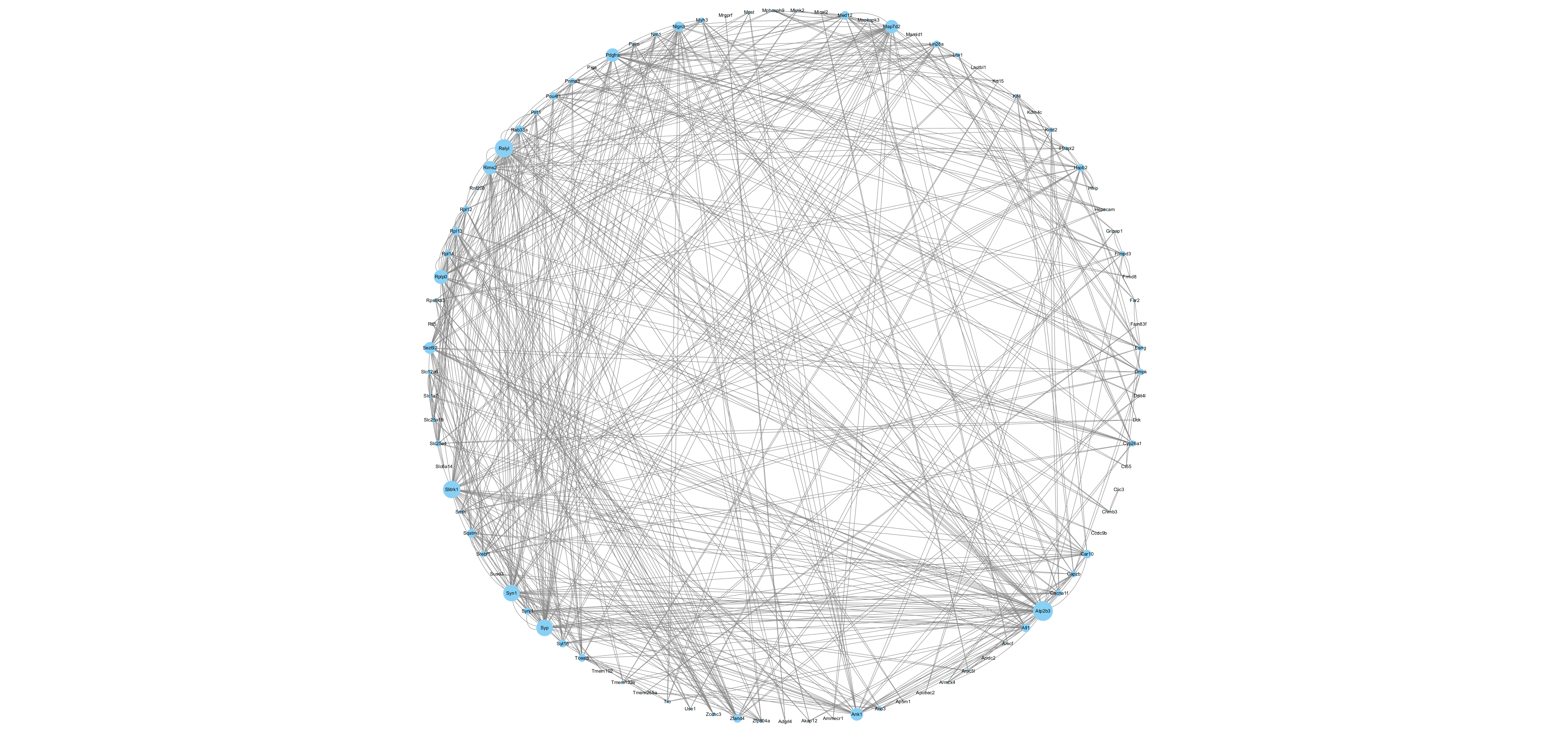

Supplement: S1 Fig — (TIF) [file pone.0316766.s001.tif]

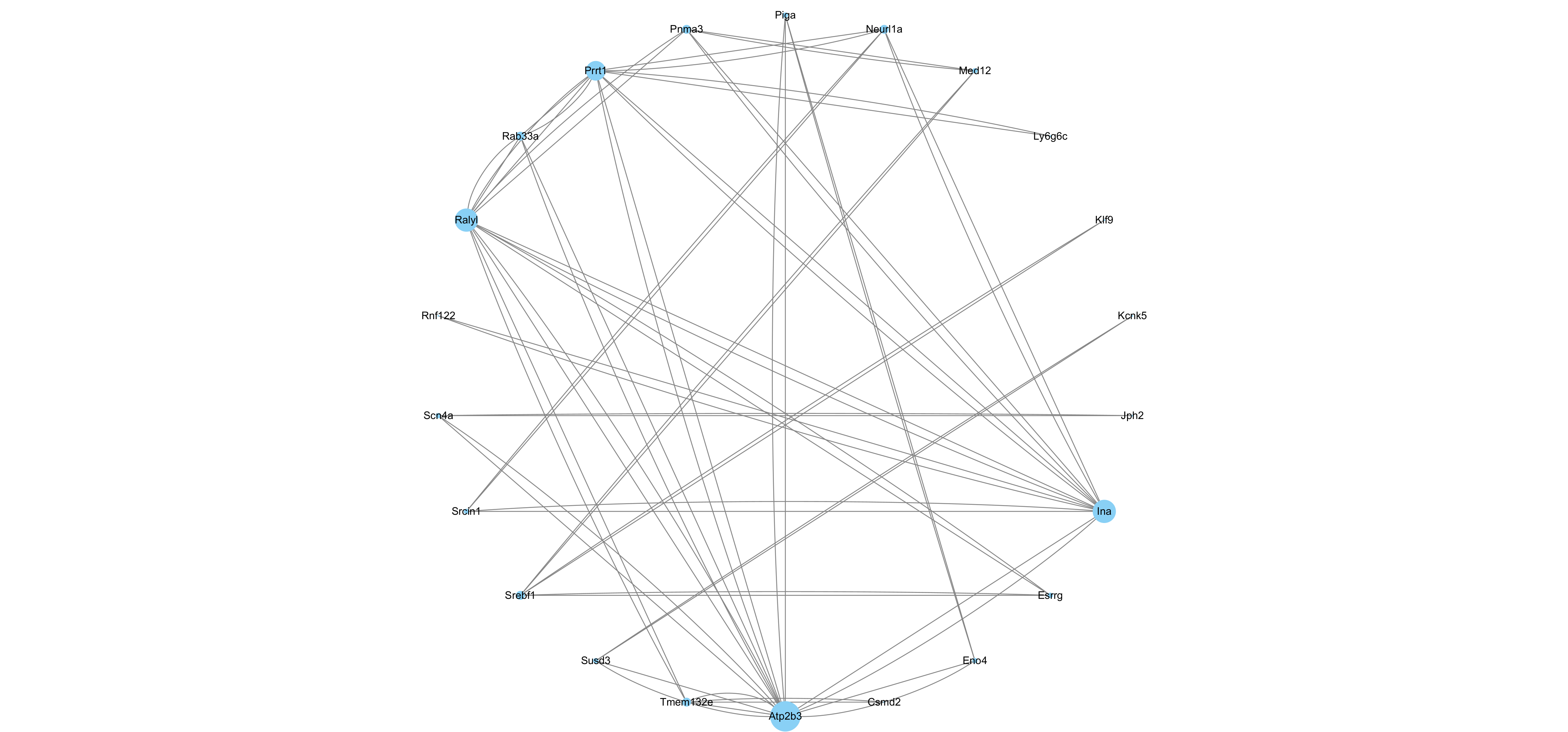

Supplement: S2 Fig — (TIF) [file pone.0316766.s002.tif]
